# Mapping online patient reviews onto a data-informed service-experience framework: a content analysis of tertiary hospitals in Wuxi, China

**DOI:** 10.3389/fpubh.2026.1838026

**Published:** 2026-07-13

**Authors:** Tiange Xia, Wenyu Tian, Linhui Li, Yijie Zhang, Yi Zhou, Shaoshuo Li, Yang Shao, Jianwei Wang

**Affiliations:** 1 Nanjing University of Chinese Medicine, Nanjing, China; 2 Wuxi TCM Hospital Affiliated to Nanjing University of Chinese Medicine, Wuxi, China; 3 Guangzhou University of Chinese Medicine, Guangzhou, China

**Keywords:** China, digital public health, hospital service experience, online patient reviews, patient experience, patient-reported experience measures, tertiary hospitals, text sentiment

## Abstract

**Background:**

Online patient reviews may provide patient-generated textual feedback on hospital service experience and may complement formal patient-reported experience measures (PREMs), but evidence from ordinary Chinese prefecture-level cities remains limited. This study examined whether online patient reviews could be systematically mapped onto a data-informed service-experience framework in tertiary hospitals in Wuxi, China.

**Methods:**

We conducted a cross-sectional content analysis of 2,381 online patient reviews from Dianping for tertiary hospitals in urban Wuxi. The extracted rating variable referred to each review's overall platform rating. Free-text comments were used to identify service-experience content behind ratings. After preliminary reading, pilot coding, clinician discussion, and framework refinement, the final five-domain coding framework was fixed before annotation. Each review was classified by main experience domain, secondary experience domain, and text sentiment. We described domain distributions and examined associations with rating levels, hospital type, time period, and campus characteristics. Factors associated with low ratings were analyzed using logistic regression with campus-clustered robust standard errors, with sensitivity analyses using mixed-effects, GEE, ordinal, and sentiment-excluded models.

**Results:**

Online patient reviews showed interpretable domain patterns within the fixed, data-informed framework. Efficiency/Process was the most common main domain, followed by Perceived Quality and Convenience/Environment. Low-rating reviews were more often centered on Attitude/Communication and accompanied by Negative or Mixed text sentiment. Compared with reviews primarily focused on Perceived Quality, reviews centered on Attitude/Communication showed stronger associations with low ratings, whereas reviews centered on Efficiency/Process or Convenience/Environment showed weaker associations. Negative and Mixed text sentiment were both strongly associated with low ratings. Contextual variables such as hospital type and parking availability were less consistent across modeling frameworks and should be interpreted cautiously.

**Conclusion:**

Online patient reviews of tertiary hospitals in Wuxi could be systematically mapped onto a fixed, data-informed five-domain framework and may provide supplementary signals for identifying recurrent perceived service frictions, particularly those related to communication and process improvement. These findings may offer complementary information for patient-centered service monitoring in similar urban tertiary-hospital contexts, but should be interpreted alongside formal PREMs, patient-experience surveys, complaint records, and hospital quality indicators.

## Introduction

With the continued development of health systems worldwide and rising demand for care, patient experience has become an important dimension in evaluating hospital quality of care, clinical safety, and management performance (1, 2). Patients' overall evaluations of hospitals depend not only on treatment outcomes themselves, but also on the experience of the care-seeking process as a whole (3, 4). In recent years, doctor-patient relations in China have remained a focus of attention (5, 6). Beyond diagnosis- and treatment-related factors, a substantial share of doctor-patient friction is closely related to experience problems arising during care processes (6–8). More timely and more authentic identification of patients' concerns and sources of dissatisfaction during care has therefore become an important issue in refined hospital management and public health governance (7, 8).

Historically, hospital managers and researchers have relied mainly on standardized surveys and multiple in-hospital feedback channels to understand patient satisfaction and experience (9–12). These tools offer institutionalized and quantifiable advantages, but they often do not fully capture the specific problems that concern patients in practice (12–14). Many experiences that do not develop into formal complaints, but already generate negative feelings, are scattered through patients' everyday expressions and are difficult for traditional management systems to capture systematically (15–17). As internet platforms have become more deeply integrated into daily life, more patients have chosen to share care experiences on public platforms, express satisfaction and dissatisfaction, and, to some extent, influence the care-seeking decisions of others (18–20). Compared with in-hospital surveys, such online reviews combine ratings with free text, are more spontaneous and contextual, and can be understood as patient-generated textual feedback reflecting hospital service experience (21–23).

In China, this perspective has particular practical relevance. China has a large patient population, marked differences across hospital tiers, and uneven allocation of medical resources across regions. Patients' evaluations of hospitals are related to diagnosis and treatment themselves, but are also jointly shaped by service organization, process arrangements, and the care environment (24–27). At the same time, internet platforms have become an important channel through which the public obtains medical information, compares hospital services, and expresses care experiences (21, 28–30). Previous studies suggest that, after human review, large language models operating within a prespecified coding framework can achieve accurate structured classification of medical free text, and that systematic checks of the logical and informational consistency of model outputs can support reliable text interpretation (31–33). Related natural language processing studies also suggest that thematic and sentiment information can be extracted from free text to some extent (17, 34–36). However, existing research has focused mainly on negative reviews, specific events, or single-hospital settings, and has paid relatively limited attention to the overall domain distribution of service experience reflected in online reviews. The fine-grained relationship between rating levels and review content remains to be clarified, and the context-dependent nature of textual expression likewise suggests that the two are not linked in a simple linear way (17, 31, 36). In addition, prior studies of patient reviews have concentrated largely on megacities or areas with concentrated medical resources, while empirical evidence from tertiary hospital systems in ordinary prefecture-level cities remains limited (31, 37).

Against this background, Wuxi offers a useful study setting. The city reflects the characteristics of a relatively affluent prefecture-level city in the Yangtze River Delta, yet differs from megacities with highly concentrated medical resources (38, 39). It is also situated in a typical context of continuing change in China's urban health service system and regional medical coordination (39–42). Studying online patient reviews of tertiary hospitals in Wuxi therefore helps supplement the existing literature with empirical evidence from the context of an ordinary prefecture-level city.

Building on this context, the present study treats online patient reviews as patient-generated experience data that may complement, but not replace, formal patient-reported experience measures (PREMs) (43). We developed a five-domain service-experience framework by combining prior patient-experience concepts, Donabedian's structure-process-outcome model, preliminary reading, pilot coding, and clinician discussion. The final framework, label definitions, and boundary rules were fixed before full-scale annotation. This study aimed to examine whether Dianping reviews could be mapped onto interpretable experience domains and whether these review-based signals could provide supplementary information for hospital service monitoring, service-friction identification, and patient-centered quality improvement in a Chinese non-megacity tertiary-hospital setting. Accordingly, this study should be understood as a framework-based mapping analysis using a data-informed but fixed coding framework, rather than an inductive thematic analysis intended to generate a new taxonomy of patient experience.

## Materials and methods

### Study design and setting

This study was a cross-sectional content analysis based on publicly available online reviews. The study population comprised user reviews of tertiary hospitals, all of which held Chinese grade-A tertiary status, and their campuses within the main urban area of Wuxi on the Dianping platform. Dianping is a widely used open commercial review platform in China that publicly displays merchant ratings, user reviews, and posting dates, and provides accessible review text and rating data for research on hospital service experience (https://www.dianping.com/; accessed February 2026). Previous studies have also used Dianping hospital reviews to analyze medical service evaluation (31). In addition, the study used point-of-interest (POI) information from Amap to standardize hospital and campus identification, determine institutional affiliation, and analyze area-level characteristics. Amap provides location search and nearby-POI display functions that can be used to extract background indicators of transport accessibility around hospitals or campuses (https://www.amap.com/; accessed February 2026). Similar studies have also used Amap POI data to analyze the spatial distribution of social facilities (44).

**Figure 1:**
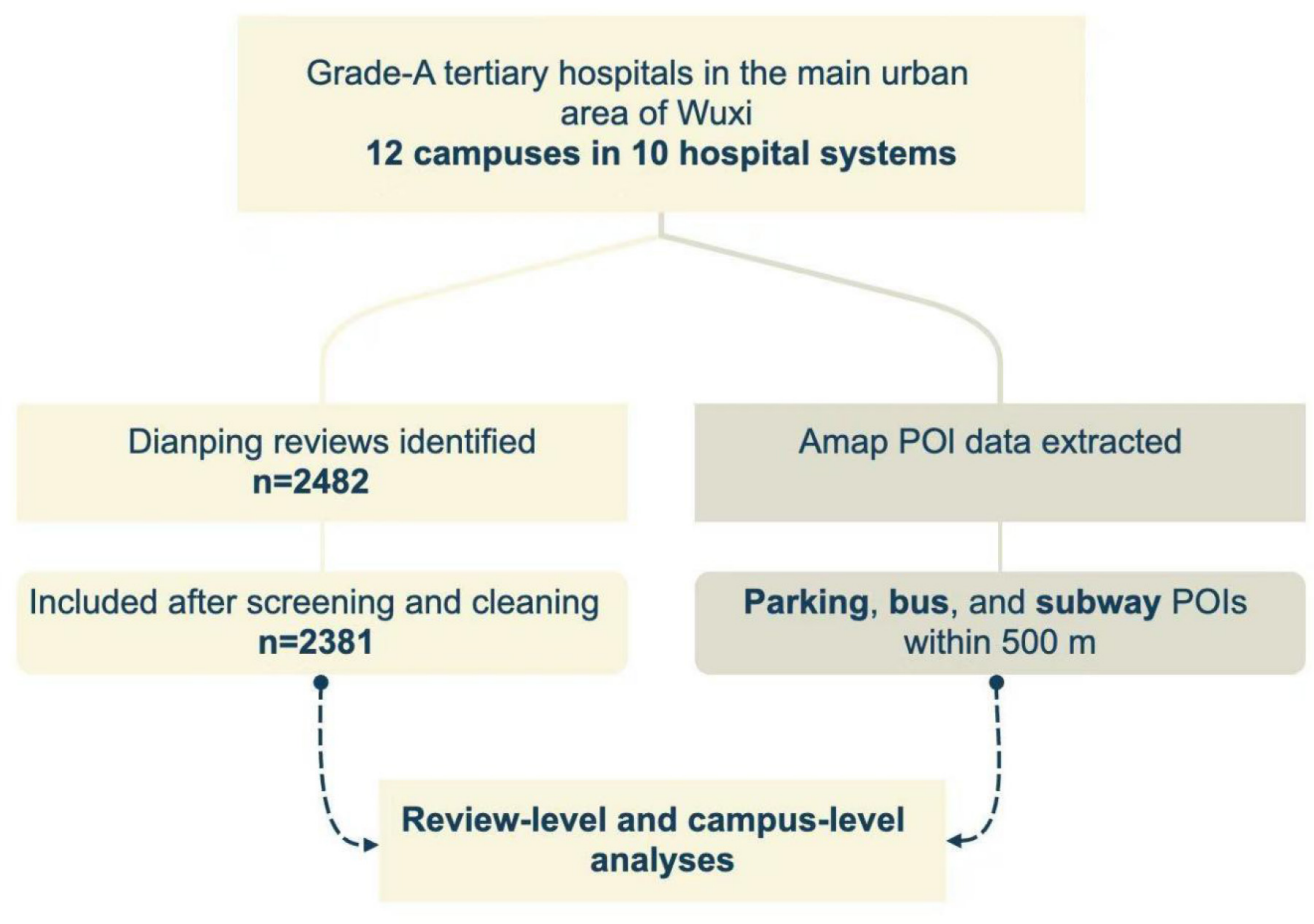
Flowchart of study setting identification, data collection, and analytic dataset construction. Grade-A tertiary hospitals and their campuses in the main urban area of Wuxi were identified first, yielding 12 campuses within 10 hospital systems. Dianping reviews were then collected and screened to obtain 2,381 eligible reviews. In parallel, Amap POI data were used to derive campus-level contextual variables within 500 m. The two data sources were merged for review-level and campus-level analyses.

To reduce the risk of institutional identification, real hospital and campus names were used only for internal data cleaning and affiliation determination; in the main text, tables, and figures, all hospitals and campuses are presented in anonymized form using study codes. The overall study workflow, including hospital identification, review screening, POI extraction, and analytic dataset construction, is summarized in Figure 1.

## Ethics

Ethical and privacy considerations, including the handling of publicly displayed usernames and de-identification procedures, are described in the Ethics statement.

## Data source and collection

Review data were obtained from Dianping.

Dianping allows users to post a free-text review together with a numerical rating for the reviewed institution. In this study, the extracted rating variable referred to the overall platform rating available for each review. Because this overall rating provides only a summary evaluation and does not indicate which part of the care experience the user was referring to, the free-text comments were important for the present analysis. They provided the basis for identifying the experience domains behind users' overall ratings.

Using standardized hospital and campus names as search terms, the research team manually searched the relevant publicly displayed storefront pages on the platform and collected store name, user name, review text, rating, and posting date. Each review was treated as an independent unit of analysis.

Inclusion criteria were as follows: the review target could be clearly matched to a hospital or campus within the study scope; the review text contained basic semantic information; and complete rating and posting-date information was available. Exclusion criteria were reviews with unclear attribution, duplicate entries, reviews consisting only of emojis or symbols, extremely short reviews with indeterminate experience content, and reviews clearly unrelated to medical service experience.

To ensure text quality, reviews with fewer than 10 total characters and fewer than 10 distinct Chinese characters were excluded. For any user ID with more than five reviews, manual verification was conducted first, and the reviews were retained only if the content was confirmed to be authentic, non-duplicate, and semantically independent. Usernames were used only for internal deduplication and quality control and are not reported in any results.

The platform rating was recorded on a 0.5–5.0 scale. For descriptive stratification and regression analysis, ratings were grouped into three categories: low rating (0.5–2.5), middle rating (3.0), and high rating (3.5–5.0). This grouping was used because 3.0 represents the midpoint of the platform scale, some original half-point rating categories contained relatively few reviews, and the main aim was to identify review content associated with clearly lower overall evaluations rather than to model each half-point score separately. To retain transparency, the full distribution of original rating categories is reported in Supplementary Table S6. Ordinal regression using the three ordered rating groups was also performed as a sensitivity analysis.

As a descriptive and adjustment strategy based on the distribution of review counts, reviews were divided into three periods according to posting date: T1 (2009–2013), T2 (2014–2017), and T3 (2018–2025). These periods were used mainly for descriptive comparison and coarse-grained temporal adjustment in the models, rather than to identify historical breakpoints related to platform development, policy change, or hospital reform. Accordingly, no strong mechanistic meaning was assigned to the time grouping.

## Hospital identification and classification

Based on the target hospital list and Amap POI information, the research team constructed a master hospital file to standardize hospital names, identify campus affiliation, extract administrative district information, and distinguish different campuses within the same hospital system. During data processing, reviews were matched to specific campuses whenever possible; multiple campuses within the same hospital system were merged only for hospital-system-level comparisons. Hospitals were further classified as general tertiary or specialty tertiary hospitals for stratified comparisons of experience-domain distributions across hospital types.

Using Amap POI information, the research team manually extracted background measures around each hospital or campus. Specifically, after searching for each target hospital or campus in Amap, the team used the platform's “Nearby” function to record the numbers of subway stations, parking facilities, and bus stops within a 500-m straight-line distance. Parking-related POI counts were used as a limited proxy for nearby parking-related facilities rather than as a direct measure of actual parking convenience or accessibility.

## Coding framework

We used a human-led, data-informed coding framework, with large language model assistance used only for classification within the fixed framework. The hospital service-experience coding manual was developed through multiple rounds of discussion based on previous studies of patient experience and online reviews (31, 47, 48, 59), Donabedian's structure-process-outcome model, the professional judgment of two senior clinicians, and preliminary reading and pilot coding of several 100 reviews. The framework was therefore not purely deductive. After pilot coding and refinement, the final five-domain framework, label definitions, boundary rules, and output format were fixed before full-scale annotation. The LLM was then used only to classify reviews within this fixed label set and did not generate new domains during full-sample coding.

Conceptually, Donabedian's structure-process-outcome model was used as a scaffold for operationalizing patient-experience content rather than as a direct three-category coding scheme (45). Convenience/Environment was treated as a structure-related domain; Attitude/Communication, Efficiency/Process, and Cost/Transparency as process-related domains; and Perceived Quality as an outcome-related domain. Cost/Transparency was retained as a separate patient-facing process-related domain because cost and billing transparency were recurrent concerns in the reviews.

The experience domains in the reviews were coded using a closed label set with five prespecified categories: Attitude/Communication, Efficiency/Process, Cost/Transparency, Perceived Quality, and Convenience/Environment. Each review was assigned one main experience-domain label, defined as the domain carrying the primary evaluative meaning of the review. A secondary experience-domain label was recorded only when the review also contained another distinct and substantively meaningful experience issue; thus, each review retained at most two experience-domain labels. For example, a review mentioning both careful explanation and long waiting time could be coded as involving Attitude/Communication and Efficiency/Process, with the main domain determined by the dominant evaluative emphasis of the review. Reviews without a clear second domain, or with only incidental or weakly evaluative mentions, were not assigned a secondary domain. Because the regression model used the presence of a secondary experience domain as a binary variable, we separately evaluated agreement for the presence or absence of a clear secondary domain and category classification among reviews judged to contain a secondary domain.

Text sentiment was classified into three categories under predefined rules: Positive, Negative, and Mixed, on the basis of the overall semantic meaning of the review rather than the platform rating. Positive sentiment indicated that the review was mainly affirmative, satisfied, or complimentary overall; Negative sentiment indicated that the review was mainly dissatisfied, critical, or complaining overall; and Mixed sentiment indicated that the review contained both clear strengths and clear weaknesses, both of which formed core content of the review. Each review retained only one text-sentiment label. If a review was too brief, contained insufficient information, or was merely factual, such that a clear evaluative direction could not be determined from the coding manual, it was not included in text-sentiment-related analyses. Distributional summaries and agreement assessments for text sentiment were conducted among reviews included in the text-sentiment analysis.

## Human annotation and LLM-assisted coding

After the coding manual had been finalized, the research team first assessed manual coding reliability in an independent formal manually coded subset of 200 reviews. This subset was drawn from the eligible review pool before full-scale LLM-assisted batch coding and was used only to assess whether human coders could apply the predefined coding manual consistently. Two researchers independently annotated the reviews, and disagreements were resolved through discussion, with adjudication by a third researcher when necessary. This assessment evaluated the reliability of applying the predefined coding manual by human coders and was distinct from the subsequent validation of LLM-assisted labels against post-adjudication human consensus labels. After full-scale LLM-assisted batch coding had been completed, a separate 200-review LLM-output validation subset was sampled from the same eligible review pool according to the LLM processing order. To ensure non-overlap, reviews already included in the human inter-rater reliability subset were excluded from the sampling frame before the LLM-output validation subset was drawn. The two subsets were therefore independent and served different methodological purposes: the former assessed human coding reliability, whereas the latter assessed agreement between initial LLM-assisted labels and post-adjudication human consensus labels. In the formal manual-coded sample, inter-rater agreement for the main experience domain was 91%, with a Cohen's kappa of 0.84. Among reviews included in the secondary-domain analysis, agreement for the secondary experience domain was 86%, with a Cohen's kappa of 0.75. Among reviews included in the text-sentiment analysis, agreement for text sentiment was 89%, with a Cohen's kappa of 0.80.

After the coding manual had been confirmed by the research team, we introduced a ChatGPT-based large language model to assist with structured coding of the large review sample. The model used in this study was GPT-5.4 Thinking, accessed in March 2026. To improve methodological transparency and reproducibility, we used a fixed structured prompt template throughout batch coding. This template was built directly from the coding manual and explicitly specified the definitions of the main experience domain, secondary experience domain, and text sentiment, together with the boundary-handling rules, exclusion scenarios, and standardized output format; the full coding manual and structured prompt template are provided in Supplementary Appendices 1, 2. The model performed structured classification only within the fixed coding framework and did not generate a new label system.

We used this three-step annotation procedure to keep the coding process interpretable and easy to review. The main experience domain was used to identify the dominant service issue in each review. The secondary domain was recorded only when a clearly distinct additional issue was present, so that major co-occurring service issues could be captured without turning short reviews into unstable multi-label annotations. Text sentiment was coded separately from the numerical rating because ratings and review narratives may not carry the same information. This approach was consistent with the aim of mapping service-experience content rather than developing an optimized natural language processing model.

For each review, the model first determined the main experience domain; if the review also clearly involved a second distinct experience domain, the model then determined a secondary experience domain; on that basis, reviews with a clearly identifiable evaluative direction were further assigned text sentiment. The model was allowed to classify only within the prespecified label set and to output results in the fixed format “main label-secondary label-text sentiment.” If a review contained both clear strengths and clear weaknesses, it was classified as Mixed. If the boundary between main and secondary labels was unclear, the experience domain corresponding to the main praise or main complaint in the review was treated as the main label. If the model could not determine a clear main experience domain under the fixed coding framework, the review was not included in experience-domain-related analyses; if it could not determine clear text sentiment, the review was not included in text-sentiment-related analyses.

For model-output validation, we used a block-based random-sample review procedure based on LLM processing order. After the eligible review dataset had been pooled for batch coding, consecutive processing blocks were constructed according to the actual LLM processing sequence rather than by hospital, campus, rating group, time period, or main experience domain. Reviews were then randomly sampled from across these consecutive blocks to obtain a 200-review validation subset, so that the validation reviews were not drawn from only one segment of the batch-coding process. After sampling, the validation subset was descriptively compared with the full analytic sample by rating group, time period, hospital type, and main experience domain; the results are provided in Supplementary Table S5. The validation subset covered the major characteristics of the full analytic sample, although modest differences were observed for some categories, such as Attitude/Communication and the 2014–2017 period. In the validation subset, initial LLM-assisted labels were compared with post-adjudication human consensus labels. Agreement/accuracy was 87.0% for main experience-domain coding, 90.0% for secondary-domain category classification, and 85.0% for text-sentiment coding. Secondary-domain validation was reported in two steps: binary presence/absence agreement and category classification in the secondary-domain category-validation subset, as shown in Supplementary Table S2. These values therefore represent agreement between initial LLM-assisted labels and post-adjudication human consensus labels, rather than human inter-rater agreement. Category-specific precision, recall, F1-score, support, and confusion matrices are reported in Supplementary Table S2. Metrics for low-frequency categories, particularly Cost/Transparency, should be interpreted cautiously because precision, recall, and F1-scores may be unstable when validation counts are small.

## Statistical analysis

Statistical analysis was conducted at two levels: the review level and the campus level. Review-level analyses included all eligible reviews and were used to describe the distributions of ratings, experience-domain labels, and text sentiment, and to compare differences in review content patterns across rating groups, hospital types, and time periods. Campus-level analyses included only anonymized campuses meeting predefined review-volume thresholds: at least 50 reviews for the main campus-level analysis, 30–49 reviews for supplementary description or sensitivity analyses, and fewer than 30 reviews excluded from campus-level comparison. Categorical variables are presented as frequencies and proportions, and between-group distributions were compared using Pearson's chi-square tests. All tests were two-sided, and *P* < 0.05 was considered statistically significant. Continuous campus-level variables are described using means and standard deviations, medians (interquartile ranges), and ranges. Data processing, descriptive statistics, cross-tabulation analyses, and visualization were conducted mainly in Python.

In the analysis of factors associated with low-rating reviews, we constructed a binary outcome variable, low_rating, coding platform ratings of 0.5–2.5 as 1 and 3.0–5.0 as 0. Time period was defined according to review year as 2009–2013, 2014–2017, and 2018–2025. Two fixed-effect models were prespecified. Model 1 included main experience domain, text sentiment, the presence of a secondary experience domain, and time period. Model 2 further adjusted for hospital type and parking availability. Here, the presence of a secondary experience domain was a binary variable indicating whether a review was judged to contain a clear second experience domain, rather than the specific category of the secondary label. Parking availability was first constructed at the campus level from the number of parking facilities and then merged back to the review level; it was dichotomized at the median campus-level parking_count_500 m. The current median threshold was 20.5; thus, low parking availability was defined as parking_count_500 m < = 20.5 and high parking availability as parking_count_500 m > 20.5. The primary analysis used review-level logistic regression with HC1 cluster-robust standard errors at the campus level to account for within-campus correlation among reviews. As sensitivity analyses, we additionally fit the same covariate structure using two alternative approaches: a mixed-effects logistic regression with a random intercept for campus, and a generalized estimating equation (GEE) logistic regression clustered by campus with an exchangeable working correlation structure. To address potential information loss from dichotomizing ratings, we performed a proportional-odds ordinal logistic regression sensitivity analysis using three ordered rating categories: low rating (0.5–2.5), middle rating (3.0), and high rating (3.5–5.0). The model used the same covariate structure as the fully adjusted primary model. Odds ratios greater than 1 indicate higher odds of being in a higher rating category. A cumulative-logit diagnostic was used to examine directional consistency across rating thresholds, and full estimates with 95% confidence intervals are reported in Supplementary Table S3. Because text sentiment and numerical ratings are conceptually overlapping evaluative constructs, a sensitivity model excluding text sentiment was also fitted and is reported in Supplementary Table S4. Effect sizes are reported as odds ratios (ORs) with 95% confidence intervals (CIs). All results were interpreted as associations rather than causal effects. Regression and sensitivity analyses were conducted in R 4.4.1.

## Results

### Sample characteristics

A total of 2,381 online patient reviews were included, covering 10 anonymized hospital systems and 12 anonymized campuses. Of these, two hospital systems each contained two campuses, whereas the remaining eight hospital systems each corresponded to a single campus. By campus review volume, there were 10 main-analysis campuses (>=50 reviews), one campus with 30–49 reviews, and one sparse campus. At the hospital-system level, four were general tertiary hospital systems and six were specialty tertiary hospital systems; at the campus level, each type included six campuses. Review dates ranged from November 22, 2009, to December 23, 2025. Ratings comprised 407 low-rating reviews (17.09%), 337 middle-rating reviews (14.15%), and 1,637 high-rating reviews (68.75%). The full distribution of the original 0.5–5.0 platform rating categories is provided in Supplementary Table S6. Among the 500-m campus-level POI indicators, the median (IQR) number of parking POIs was 20.5 (19.5, 41.0) (Table 1).

**Table 1:** Sample characteristics and analytic structure.

Section	Item	Value
Sample characteristics	Total reviews, n	2,381
	Anonymous hospital systems, *n*	10
	Anonymous campuses, *n*	12
	Main-analysis campuses (≥50 reviews), n	10
	Borderline campuses (30–49 reviews), n	1
	Sparse campuses (< 30 reviews), *n*	1
Hospital type	General tertiary hospital systems, *n*	4
	Specialty tertiary hospital systems, *n*	6
	General tertiary campuses, *n*	6
	Specialty tertiary campuses, *n*	6
Time span	Review time range	2009-11-22 to 2025-12-23
Rating groups	Low rating (0.5–2.5), *n* (%)	407 (17.09%)
	Middle rating (3.0), *n* (%)	337 (14.15%)
	High rating (3.5–5.0), *n* (%)	1,637 (68.75%)
500-m contextual POIs	Parking POIs within 500 m (per campus)	median (IQR) 20.5 (19.5, 41.0)
	Bus stop POIs within 500 m (per campus)	median (IQR) 6.0 (3.8, 8.5)
	Metro station POIs within 500 m (per campus)	median (IQR) 0.5 (0.0, 1.0)

Low-, middle-, and high-rating groups were defined as ratings of 0.5–2.5, 3.0, and 3.5–5.0, respectively. POI values are presented as median (interquartile range).

### Overall distribution

**Figure 2:**
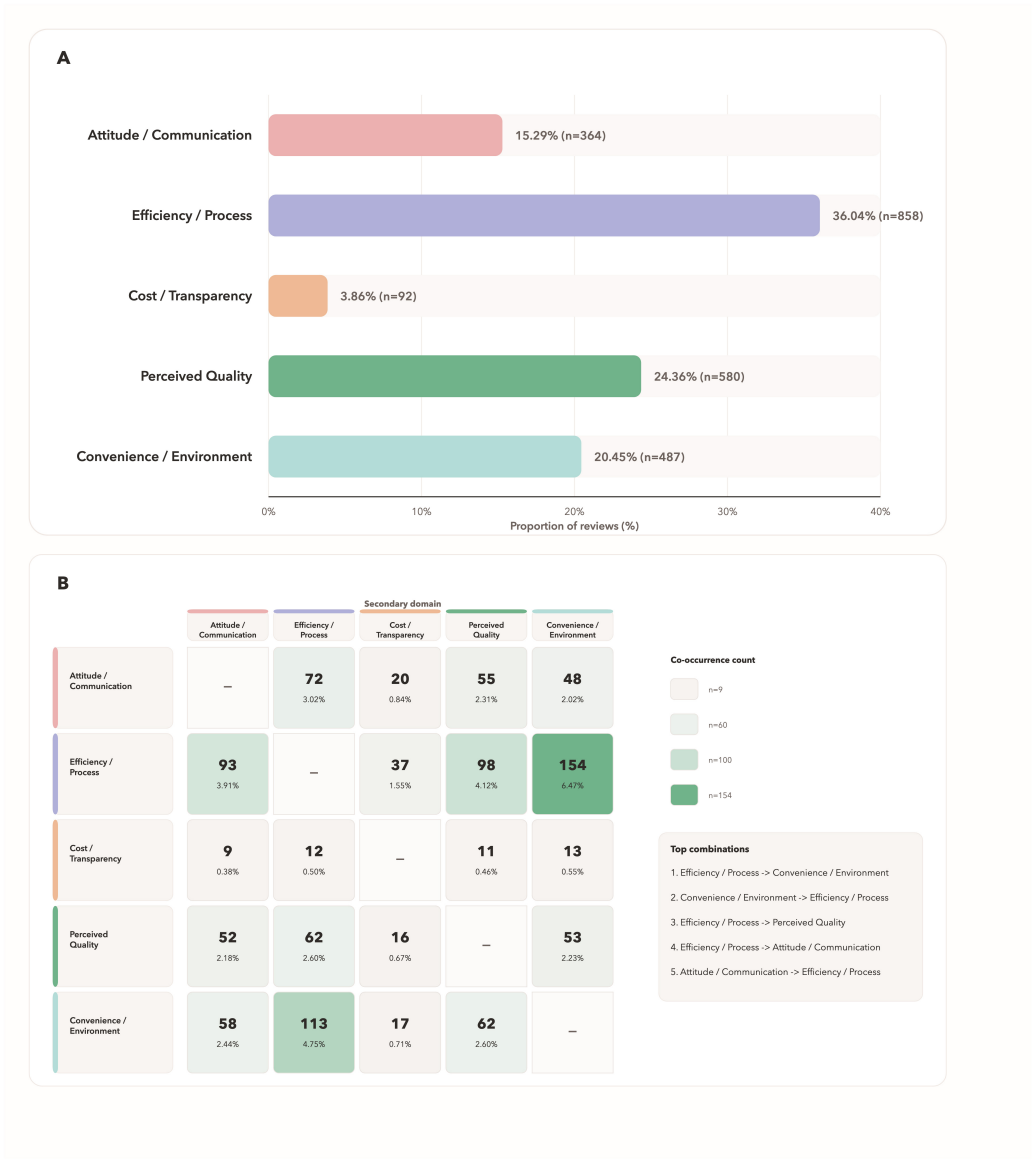
Overall distribution and co-occurrence of patient-experience domains. Panel **A** shows the overall distribution of the five main experience domains across all reviews. Panel **B** shows the co-occurrence of main and secondary experience domains among reviews assigned a secondary experience domain.

Across all reviews, the most common main experience domain was Efficiency/Process (36.04%, *n* = 858), followed by Perceived Quality (24.36%, *n* = 580) and Convenience/Environment (20.45%, *n* = 487). Attitude/Communication accounted for 15.29% (*n* = 364), whereas Cost/Transparency was least common (3.86%, *n* = 92; Figure 2A). The descriptive domain distributions reflect the content of Dianping reviews included in this sample and should not be interpreted as population-level estimates of patient-experience problems among all patients.

The co-occurrence patterns of main and secondary labels indicated that multidimensional experiences did not occur in isolation. Among reviews with a clearly identified secondary experience domain, the most common main-to-secondary pairing was Efficiency/Process to Convenience/Environment (*n* = 154), followed by Convenience/Environment to Efficiency/Process (*n* = 113) and Efficiency/Process to Perceived Quality (*n* = 98; Figure 2B).

### Differences by rating group

**Figure 3:**
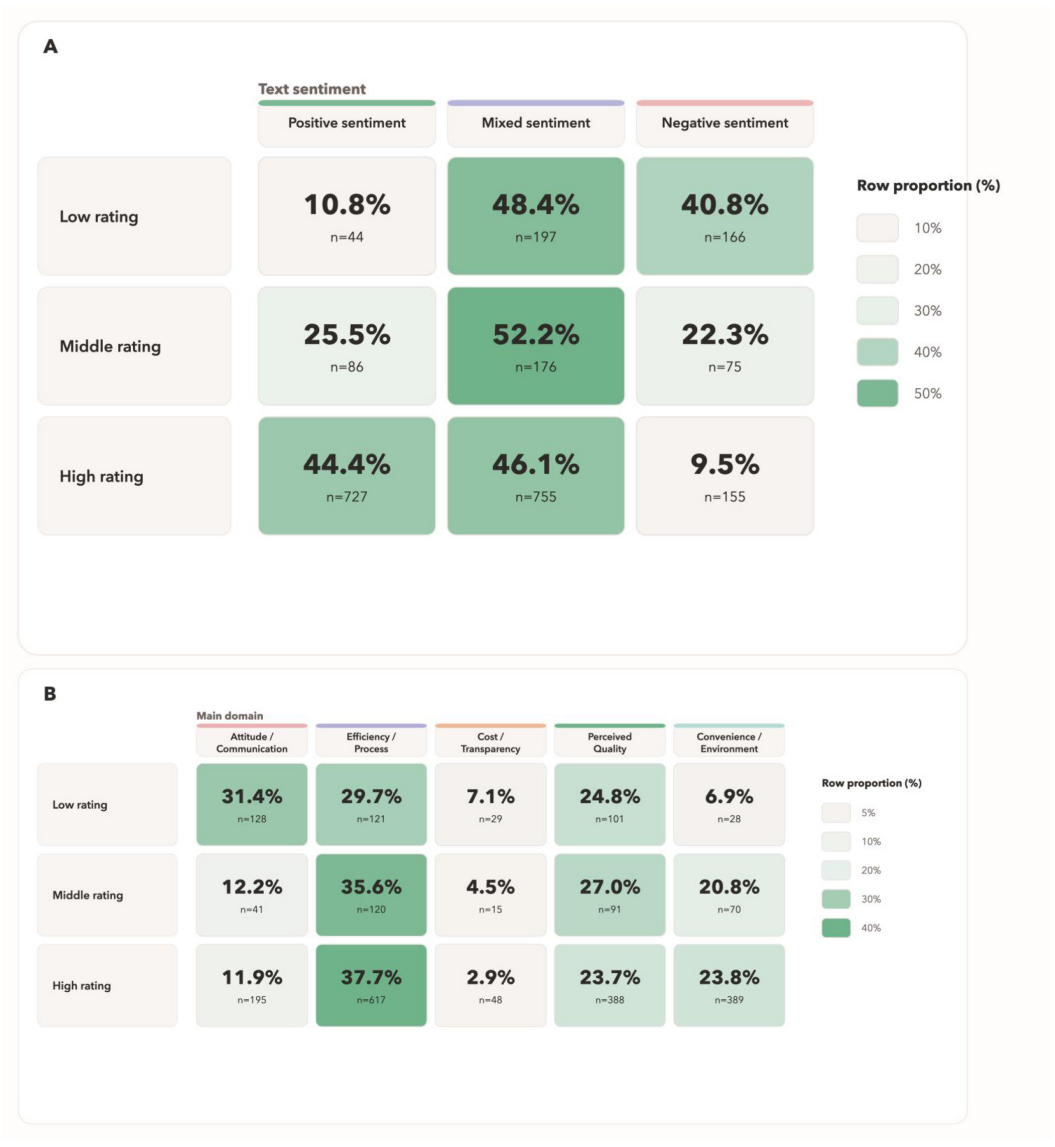
Review content patterns by rating group. Panel **A** shows the distribution of text sentiment across rating groups. Percentages were calculated using all reviews included in sentiment analysis within each rating group. Panel **B** shows the distribution of main experience domains across rating groups.

The distribution of text sentiment differed significantly across rating groups (Table 2, Panel A). Among low-rating reviews, Mixed sentiment accounted for 48.4% (*n* = 197), Negative sentiment for 40.8% (*n* = 166), and Positive sentiment for 10.8% (*n* = 44; Figure 3A). Among high-rating reviews, Mixed sentiment still accounted for 46.1% (*n* = 755), Positive sentiment for 44.4% (*n* = 727), and Negative sentiment for 9.5% (*n* = 155; Figure 3A).

**Table 2:** Distribution of review content patterns by rating group, hospital type, and time period.

Panel A. By rating group					
Category	Item	Low rating (*n* = 407)	Middle rating (*n* = 337)	High rating (*n* = 1,637)	*P* value
Main experience domain	Attitude/Communication	128 (31.4%)	41 (12.2%)	195 (11.9%)	< 0.001
	Efficiency/Process	121 (29.7%)	120 (35.6%)	617 (37.7%)	
	Cost/Transparency	29 (7.1%)	15 (4.5%)	48 (2.9%)	
	Perceived Quality	101 (24.8%)	91 (27.0%)	388 (23.7%)	
	Convenience/Environment	28 (6.9%)	70 (20.8%)	389 (23.8%)	
Text sentiment	Positive	44 (10.8%)	86 (25.5%)	727 (44.4%)	< 0.001
	Negative	166 (40.8%)	75 (22.3%)	155 (9.5%)	
	Mixed	197 (48.4%)	176 (52.2%)	755 (46.1%)	
Number of experience domains	Single domain	253 (62.2%)	203 (60.2%)	870 (53.1%)	< 0.001
	Two domains	154 (37.8%)	134 (39.8%)	767 (46.9%)	
Panel B. By hospital type					
Category	Item	General tertiary (*n* = 1,313)	Specialty tertiary (*n* = 1,068)		*P* value
Main experience domain	Attitude/Communication	207 (15.8%)	157 (14.7%)		0.002
	Efficiency/Process	487 (37.1%)	371 (34.7%)		
	Cost/Transparency	62 (4.7%)	30 (2.8%)		
	Perceived Quality	323 (24.6%)	257 (24.1%)		
	Convenience/Environment	234 (17.8%)	253 (23.7%)		
Text sentiment	Positive	475 (36.2%)	382 (35.8%)		0.542
	Negative	227 (17.3%)	169 (15.8%)		
	Mixed	611 (46.5%)	517 (48.4%)		
Rating groups	Low rating	261 (19.9%)	146 (13.7%)		< 0.001
	Middle rating	203 (15.5%)	134 (12.5%)		
	High rating	849 (64.7%)	788 (73.8%)		
Panel C. By time period					
Category	Item	2009–2013 (*n* = 160)	2014–2017 (*n* = 738)	2018–2025 (*n* = 1,483)	*P* value
Main experience domain	Attitude/Communication	30 (18.8%)	94 (12.7%)	240 (16.2%)	< 0.001
	Efficiency/Process	31 (19.4%)	242 (32.8%)	585 (39.4%)	
	Cost/Transparency	17 (10.6%)	38 (5.1%)	37 (2.5%)	
	Perceived Quality	59 (36.9%)	193 (26.2%)	328 (22.1%)	
	Convenience/Environment	23 (14.4%)	171 (23.2%)	293 (19.8%)	
Text sentiment	Positive	48 (30.0%)	264 (35.8%)	545 (36.7%)	0.444
	Negative	33 (20.6%)	123 (16.7%)	240 (16.2%)	
	Mixed	79 (49.4%)	351 (47.6%)	698 (47.1%)	
Rating groups	Low rating	47 (29.4%)	118 (16.0%)	242 (16.3%)	< 0.001
	Middle rating	53 (33.1%)	193 (26.2%)	91 (6.1%)	
	High rating	60 (37.5%)	427 (57.9%)	1,150 (77.5%)	

Panel A shows the distributions of main experience domains, text sentiment, and single-label vs. dual-label patterns across rating groups. Panel B shows the corresponding distributions across hospital-type groups. Panel C shows the corresponding distributions across time periods. Percentages for main experience domains and text sentiment were calculated using all included reviews within each comparison group. Single-label vs. dual-label pattern refers to the binary indicator of whether a review was assigned a secondary experience domain. Data are presented as *n* (%). *P* values were calculated using Pearson's chi-square test.

The composition of main experience domains also varied across rating groups (Table 2, Panel A; Figure 3B). Low-rating reviews were more concentrated in Attitude/Communication (31.4%, *n* = 128) and Efficiency/Process (29.7%, *n* = 121), whereas Convenience/Environment accounted for a smaller share (6.9%, *n* = 28; Figure 3B). At the same time, the dual-label pattern defined by the presence of a secondary experience domain accounted for 46.9% of high-rating reviews, higher than the 37.8% observed in low-rating reviews (Table 2, Panel A).

### Differences by hospital type and time

**Figure 4:**
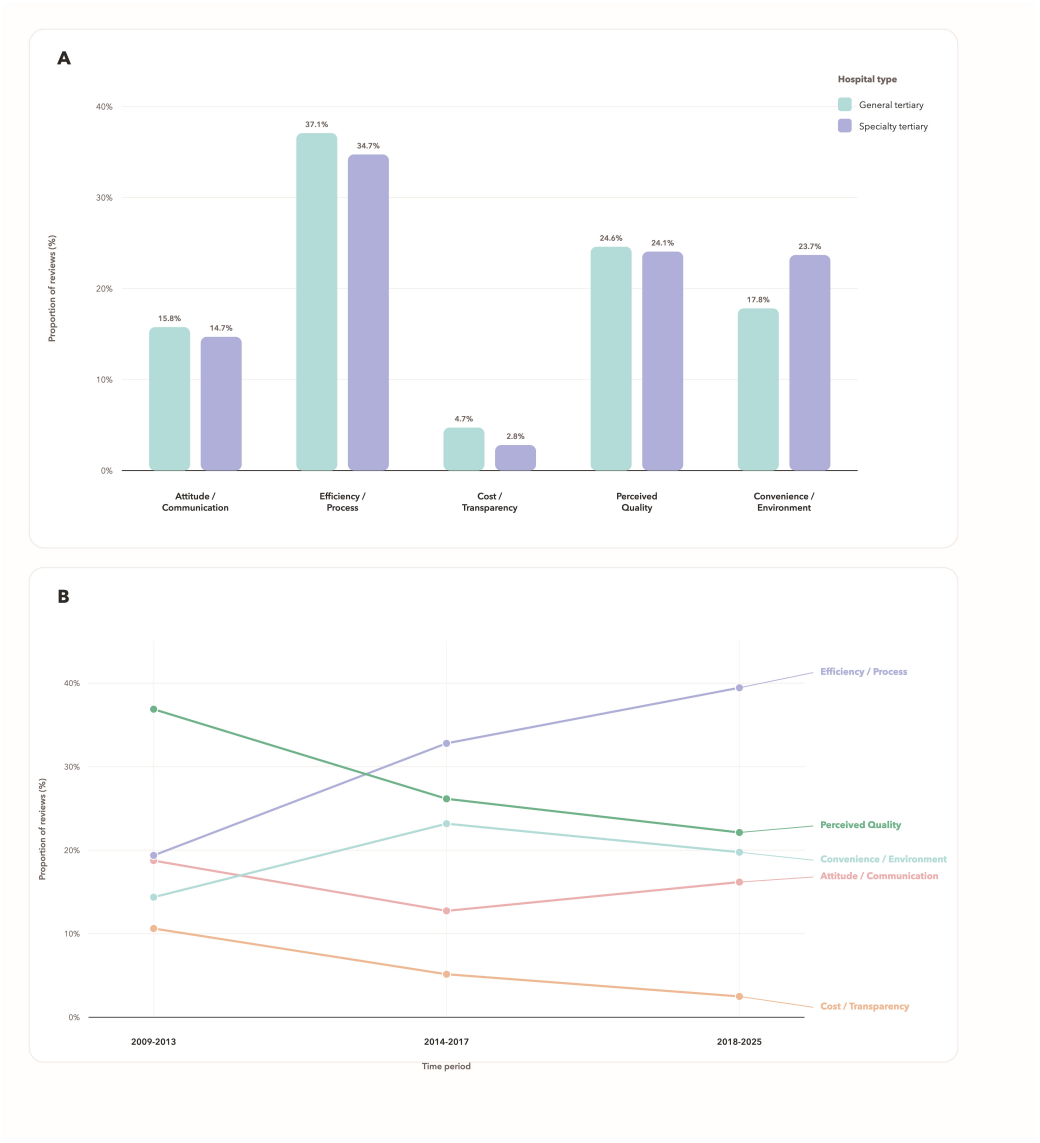
Main-domain distribution by hospital type and time period. Panel **A** compares the distribution of main experience domains between the two hospital-type groups. Panel **B** shows changes in the distribution of main experience domains across time periods.

Comparisons by hospital type showed that the general tertiary and specialty tertiary groups had broadly similar main experience-domain distributions, with Efficiency/Process ranking first in both groups (37.1% vs. 34.7%, respectively; Figure 4A; Table 2, Panel B). Convenience / Environment accounted for a higher proportion in the specialty tertiary group (23.7% vs. 17.8%; Figure 4A). Differences in text sentiment between the two hospital-type groups were not pronounced (Table 2, Panel B).

Across time periods, the distribution of main experience domains changed over time (Figure 4B; Table 2, Panel C). In 2009–2013, Perceived Quality was the leading domain (36.9%), whereas Efficiency/Process accounted for 19.4%. In 2014–2017, Efficiency/Process increased to 32.8% and exceeded Perceived Quality (26.2%). In 2018–2025, Efficiency/Process further increased to 39.4%, whereas Perceived Quality declined to 22.1% (Figure 4B). Over the same periods, the proportion of Cost/Transparency decreased from 10.6 to 5.1 to 2.5% (Figure 4B).

### Campus-level heterogeneity

**Figure 5:**
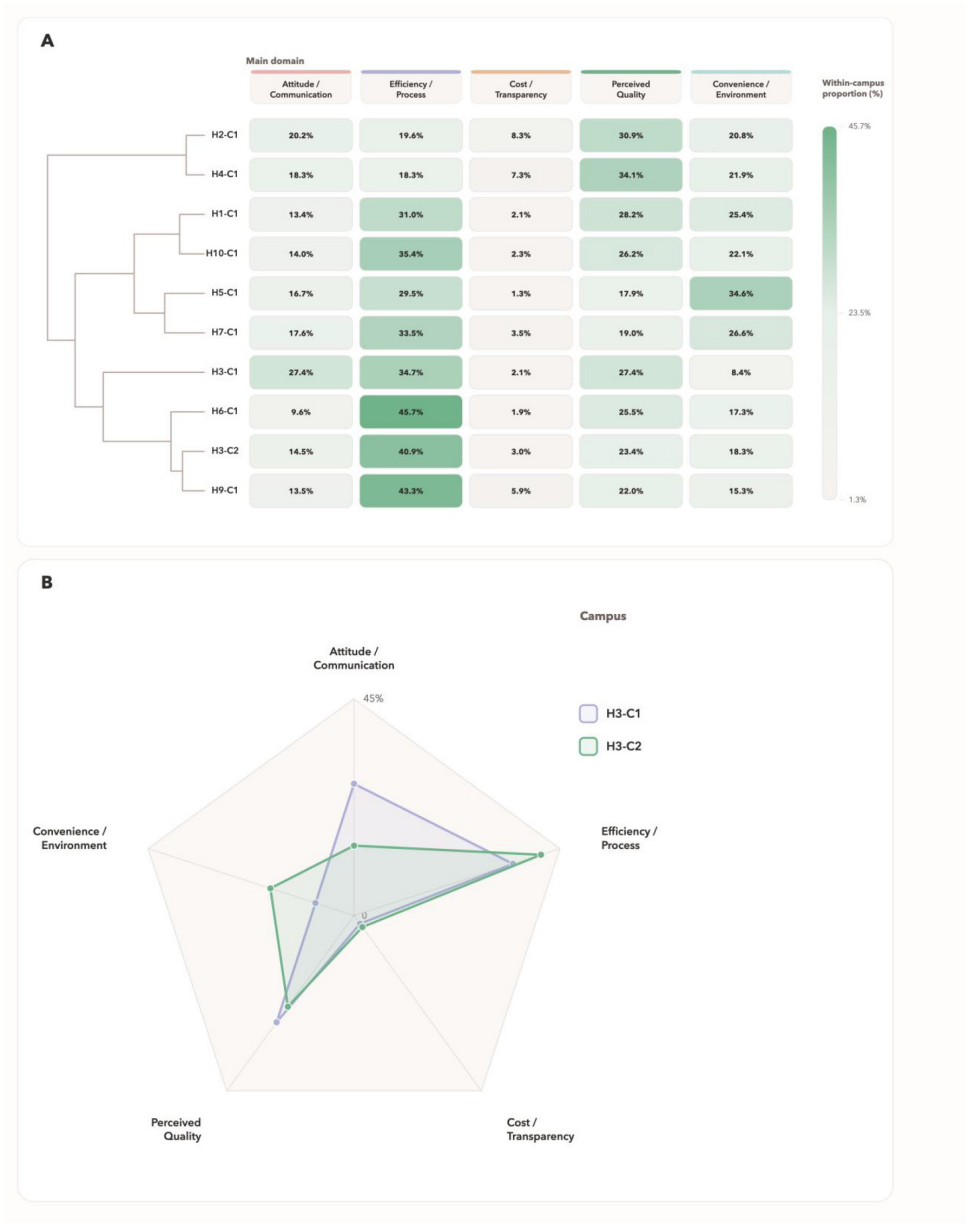
Campus-level heterogeneity in patient-experience profiles. Panel **A** shows the distribution of the five main experience domains across main-analysis campuses. Panel **B** compares representative campuses within the same hospital system.

Among the 10 main-analysis campuses, Efficiency/Process ranked first in most campuses, but the five-domain distributions showed clear heterogeneity (Figure 5A). For example, Efficiency/Process accounted for 45.7% in H6-C1, Convenience/Environment accounted for 34.6% in H5-C1, Perceived Quality accounted for 34.1% in H4-C1, and both Attitude/Communication and Perceived Quality accounted for 27.4% in H3-C1 (Figure 5A). Different campuses within the same hospital system could also show distinct profiles. Using H3 as an example, H3-C1 and H3-C2 differed in their five-domain distributions (Figure 5B).

**Figure 6:**
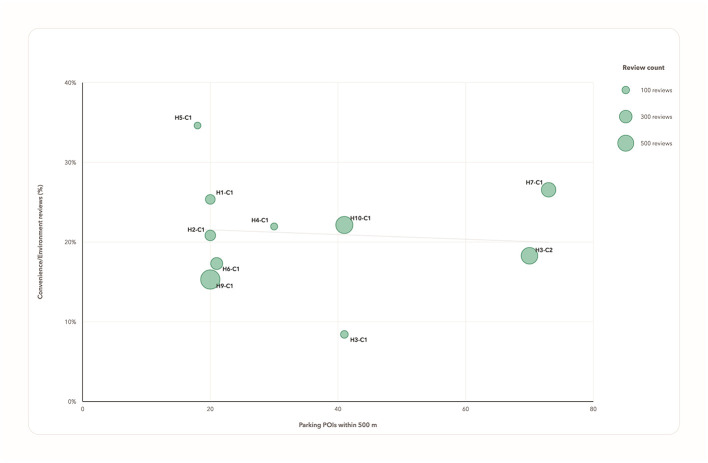
Parking availability and convenience/environment-related reviews at the campus level. Each bubble represents one main-analysis campus. Bubble size reflects the number of reviews. The figure shows the campus-level correspondence between parking availability and the proportion of reviews classified as Convenience/Environment.

At the campus level, the visual correspondence between parking availability and the proportion of Convenience / Environment-related reviews did not show a clear linear pattern in a single direction (Figure 6). Campuses with similar numbers of parking POIs could still differ substantially in the proportion of Convenience / Environment reviews, and differences were also observed between campuses within the same hospital system (Figure 6).

### Factors associated with low rating

**Figure 7:**
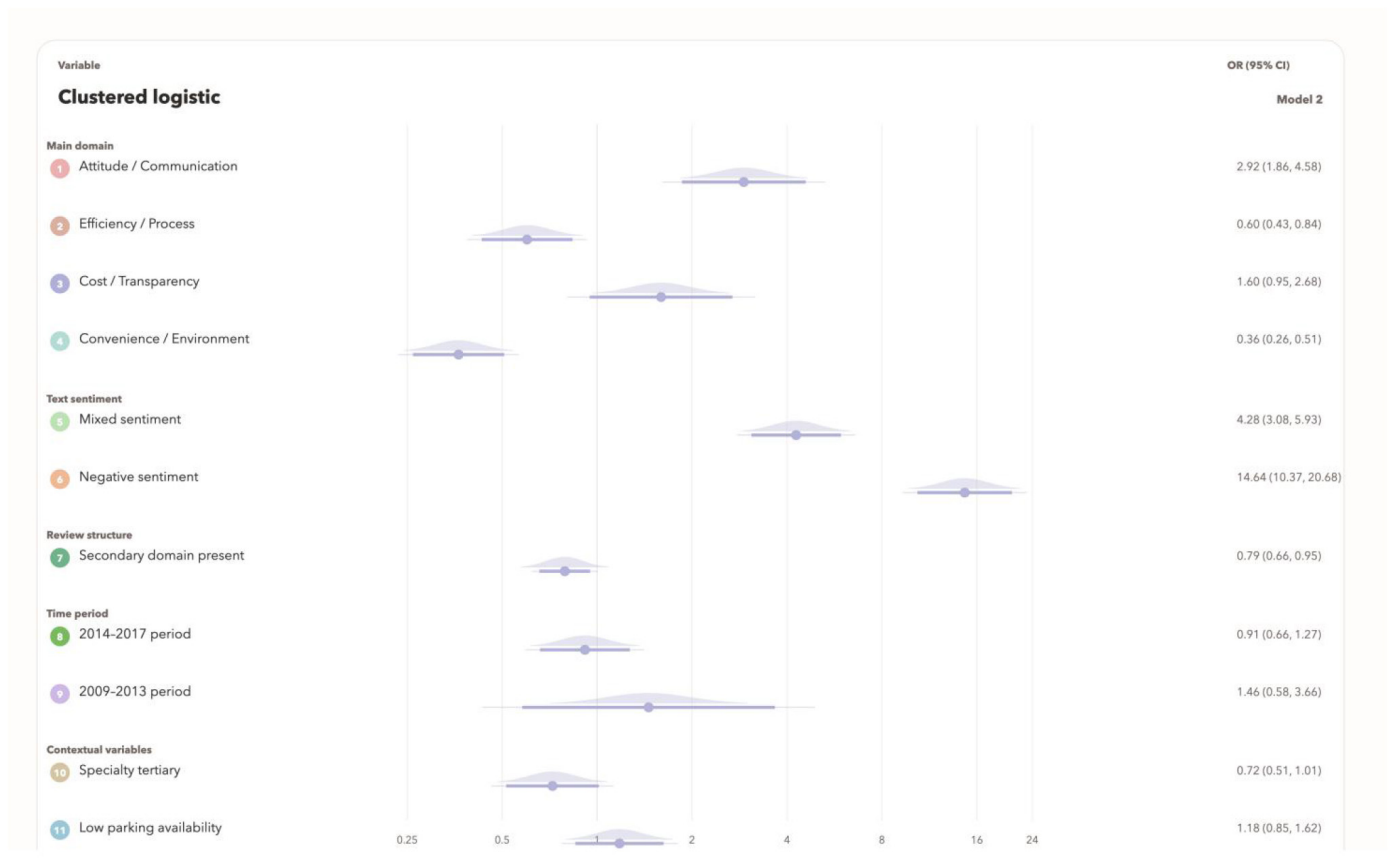
Factors associated with low rating (Model 2). Forest plot of factors associated with low_rating in the fully adjusted primary analysis model. Points represent odds ratios, and horizontal lines represent 95% confidence intervals. Odds ratios greater than 1 indicate higher odds of a low-rating review. All results should be interpreted as associations rather than causal effects.

In the primary analysis, associations between the core review-level variables and low_rating were generally consistent after adjustment for contextual covariates (Table 3; Figure 7). In the fully adjusted model, compared with reviews primarily involving Perceived Quality, reviews primarily involving Attitude / Communication were associated with higher odds of low_rating (OR = 2.92, 95% CI: 1.86–4.58, *P* < 0.001), whereas reviews primarily involving Efficiency/Process (OR = 0.60, 95% CI: 0.43–0.84, *P* = 0.002) and Convenience/Environment (OR = 0.36, 95% CI: 0.26–0.51, *P* < 0.001) were associated with lower odds of low_rating. Cost/Transparency showed a positive trend, but the estimate was relatively imprecise (OR = 1.60, 95% CI: 0.95–2.68, *P* = 0.079).

**Table 3:** Primary analysis of factors associated with low rating.

Variable	Model 1 OR (95% CI)	*P* value	Model 2 OR (95% CI)	*P* value
Main experience domain				
Attitude/Communication	2.90 (1.86, 4.53)	< 0.001	2.92 (1.86, 4.58)	< 0.001
Efficiency/Process	0.61 (0.43, 0.87)	0.006	0.60 (0.43, 0.84)	0.002
Cost/Transparency	1.67 (0.98, 2.85)	0.060	1.60 (0.95, 2.68)	0.079
Convenience/Environment	0.36 (0.26, 0.50)	< 0.001	0.36 (0.26, 0.51)	< 0.001
Text sentiment				
Mixed sentiment	4.18 (3.05, 5.74)	< 0.001	4.28 (3.08, 5.93)	< 0.001
Negative sentiment	14.31 (10.23, 20.03)	< 0.001	14.64 (10.37, 20.68)	< 0.001
Review content pattern				
Secondary domain present	0.80 (0.67, 0.96)	0.017	0.79 (0.66, 0.95)	0.012
Time period				
2014–2017 period	0.96 (0.70, 1.32)	0.796	0.91 (0.66, 1.27)	0.594
2009–2013 period	1.70 (0.70, 4.12)	0.238	1.46 (0.58, 3.66)	0.424
Contextual variables				
Specialty tertiary			0.72 (0.51, 1.01)	0.059
Low parking availability			1.18 (0.85, 1.62)	0.320

The outcome variable was low_rating, coded as 1 for ratings of 0.5–2.5 and 0 for ratings of 3.0–5.0. Model 1 included main experience domain, text sentiment, the binary indicator of the presence of a secondary experience domain, and time period. Model 2 further adjusted for hospital type and parking availability. The primary analysis used review-level logistic regression with robust standard errors clustered by campus. OR > 1 indicates higher odds of a low-rating review. Reference categories were Perceived Quality, positive sentiment, no secondary experience domain, T3 (2018–2025), general tertiary, and high parking availability, respectively.

Compared with Positive sentiment, both Mixed sentiment (OR = 4.28, 95% CI: 3.08–5.93, *P* < 0.001) and Negative sentiment (OR = 14.64, 95% CI: 10.37–20.68, *P* < 0.001) were associated with higher odds of low_rating. Reviews judged to have a secondary experience domain were associated with lower odds of low_rating (OR = 0.79, 95% CI: 0.66–0.95, *P* = 0.012). Neither time-period indicator reached statistical significance after adjustment.

For contextual covariates, the specialty tertiary group showed an overall tendency toward lower odds of low_rating than the general tertiary group, but this association was only marginally significant in the primary analysis (OR = 0.72, 95% CI: 0.51–1.01, *P* = 0.059). Low parking availability was not significantly associated with low_rating (OR = 1.18, 95% CI: 0.85–1.62, *P* = 0.320). Supplementary Table S1 shows that, across logistic regression with campus-clustered robust standard errors, mixed-effects logistic regression, and GEE models, the direction and magnitude of the core review-level variables were generally consistent. Attitude/Communication, Efficiency/Process, Convenience/Environment, and sentiment-related variables showed the most consistent estimates across statistical models. By contrast, contextual variables and Cost/Transparency were more sensitive to model specification and should be interpreted with greater caution. The ordinal regression sensitivity analysis showed that the main experience-domain estimates were broadly consistent in direction with the primary binary model when accounting for the reversed interpretation of higher vs. lower ratings; full ORs and 95% CIs are reported in Supplementary Table S3. After text sentiment was removed, the main experience-domain associations remained broadly similar in direction, although some estimates changed in magnitude or statistical significance, suggesting partial conceptual overlap between sentiment and numerical rating without fully accounting for the observed domain-rating associations (Supplementary Table S4).

Overall, the three model frameworks showed broadly similar patterns at the review level, whereas campus-level contextual variables are best interpreted cautiously and mainly as background-adjustment variables.

## Discussion

### Principal findings

We conducted a cross-sectional content analysis of 2,381 online patient reviews of tertiary hospitals in Wuxi. The findings suggest that these public reviews could be systematically mapped onto a fixed, data-informed five-domain framework of hospital service experience. Reviews most often involved Efficiency / Process, followed by Perceived Quality and Convenience/Environment, whereas Attitude/Communication and Cost/Transparency accounted for smaller shares. Co-occurrence analysis further suggested that these experience domains were not independent of one another, with Efficiency/Process and Convenience/Environment frequently appearing together.

From the perspective of PREMs and patient-centered care, online reviews should be understood as a supplementary source of patient-experience information rather than as a replacement for formal measurement tools (43). Formal PREMs are designed instruments, whereas online reviews are self-initiated public comments and are influenced by who chooses to post, how the platform is used, and how patients express themselves in public. Their value lies in the details of patients' own descriptions, especially when these descriptions point to service problems that may not appear clearly in overall ratings or routine surveys. This is why free-text online reviews may be useful when interpreted together with formal PREMs, complaint records, and hospital quality indicators.

The fixed five-domain mapping can also be read in relation to Donabedian's structure-process-outcome model. Convenience / Environment reflected structure-related cues visible to patients; Attitude/Communication, Efficiency/Process, and Cost/Transparency reflected process-related service frictions; and Perceived Quality reflected patient-perceived outcome-related evaluation. These categories represent patient-side review signals mapped onto the Donabedian framework, rather than direct objective measurements of hospital quality.

When considered together with ratings, low-rating reviews were more concentrated in narratives centered on Attitude/Communication and were also more often accompanied by negative or mixed text sentiment. In the fully adjusted review-level model, compared with reviews primarily focused on Perceived Quality, reviews centered on Attitude/Communication were more likely to be associated with low ratings, whereas reviews centered on Efficiency/Process or Convenience/Environment showed weaker associations. Mixed and negative sentiment showed the strongest associations with low ratings, suggesting that text sentiment itself represents an important layer of review information (9, 36, 46). Text sentiment is better understood as an evaluative layer of the review text that partially overlaps with the numerical rating, rather than as an independent causal determinant of low ratings. In particular, mixed sentiment may reflect care episodes in which patients acknowledged some positive aspects of care while still expressing dissatisfaction with specific frictions.

Overall, online patient reviews can, to some extent, help identify recurring perceived problems in hospital service experience and show interpretable domain patterns within a fixed, data-informed framework (47, 48). Such patient-generated review signals may be informative, but they should complement formal patient-experience surveys, complaint records, and hospital quality indicators rather than replace them (2, 48, 49).

### Interpretation in the context of tertiary hospital care

The main patterns observed in this study are broadly consistent with the care context of tertiary hospitals in China (50, 51). Patients' care experiences are related not only to whether medical care itself is perceived as adequate, but also to how care is organized and communicated (2, 50, 52). Tertiary hospitals typically involve many steps, long pathways, and frequent transitions between nodes. Registration, waiting, examinations and tests, payment and medication dispensing, and in-hospital referral may each become a source of friction in the care experience (50–52). During these processes, patients often need to interpret information continuously, wait for arrangements, and judge the messages conveyed by staff behavior under uncertainty (52–54). In this context, problems in attitude and communication may be closely related to a negative overall impression. An interaction perceived as indifferent, an unclear explanation of subsequent procedures, or an insufficient response to a patient's questions may be reflected in the patient's overall evaluation of the care episode (52, 53). Communication does not only transmit medical information, but is also part of how patients judge whether they feel respected, heard, and reassured under uncertainty. Process delays may sometimes be attributed to crowding or system-level constraints, whereas poor explanation, impatience, or perceived indifference may be interpreted more directly as insufficient professional responsiveness. The regression results were broadly consistent with this interpretation: even after adjustment for text sentiment, the presence of a secondary experience domain, and time period, Attitude/Communication remained the experience domain most strongly associated with low ratings.

By contrast, although Efficiency/Process and Convenience/Environment were high-frequency themes in online reviews, they did not show the same strong correspondence with low ratings. This suggests that process and environment issues are common in patient narratives but are not necessarily the issues most directly tied to negative overall judgments. One reasonable interpretation is that Efficiency/Process and Convenience/Environment more often function as background experiences within care episodes. They may accompany negative experiences, but they may also appear in narratives that are overall acceptable or even relatively satisfactory, whereas concerns about Perceived Quality may more readily become an important basis for patients' negative overall judgments (50–52). The association between the presence of a secondary experience domain and lower odds of low ratings should be interpreted cautiously. One possible interpretation is that the presence of a secondary domain may indicate more elaborated or mixed evaluations, whereas strongly negative reviews may focus on one dominant complaint. Accordingly, the presence of a secondary domain should be interpreted as a marker of review content complexity, rather than as evidence that multidimensional experience content reduces dissatisfaction.

### Ratings, review text, and mixed sentiment

Another notable finding was that high ratings did not necessarily mean that the review text was entirely positive. Nearly half of the high-rating reviews were classified as mixed sentiment, and some high-rating reviews still contained clear negative expressions despite high scores. This suggests that ratings and review text are not interchangeable forms of information (47, 48, 55). Ratings are closer to a compressed overall judgment, whereas text allows patients to record more detailed experiential information and concrete contexts (47, 56). Particularly in the setting of Chinese tertiary hospitals, some patients may give high ratings because they recognize physicians' technical competence, are satisfied with treatment outcomes, or wish to express gratitude, while still pointing out long waiting times, cumbersome procedures, and insufficient communication in the text (50, 52). In other words, patients may overall approve of the care episode without believing that the entire service process was frictionless. For hospital service monitoring, such information is not irrelevant noise, but one of the key values of online patient reviews. It may signal service frictions that are not yet serious enough to trigger formal complaints, but are already shaping patient experience and public perception (11, 47, 56, 57).

### Contextual variables and cross-level interpretation

Variables such as hospital type and parking availability require especially cautious interpretation because these indicators were measured at the hospital-system or campus level and then mapped back into review-level models. They are therefore better understood as contextual background factors used for adjustment, rather than as independent review-level drivers. In the fully adjusted model, the specialty tertiary group showed a tendency toward lower odds of low ratings than the general tertiary group, but this result was only marginally significant and showed some sensitivity across robustness-analysis frameworks. It may therefore be more likely to reflect background differences related to care settings than an independent effect of hospital type itself. For parking availability, the model did not observe a significant association between the number of parking-related POIs within 500 m and low ratings; the campus-level visualization likewise did not show a clear linear correspondence between parking resources and the proportion of Convenience/Environment reviews. This suggests that perceived parking difficulty or inconvenience may not be captured by the number of parking-related POIs alone, and may also involve factors not directly measured here, including parking capacity, fees, occupancy, walking distance, on-site management conditions, wayfinding systems, congestion during peak care-seeking hours, and the match between parking supply and demand. Methodologically, although this study used campus-clustered robust standard errors and further compared mixed-effects logistic regression and generalized estimating equation models to address within-campus correlation as far as possible, residual clustering and cross-level confounding remain difficult to exclude completely. These results should therefore be interpreted as review-level associations under contextual adjustment, with campus- and hospital-level variables serving mainly as background calibration.

## Implications for service improvement

The findings also suggest how online patient reviews may be used as supplementary review-based signals for hospital service improvement. In this study, such signals refer to patient-generated, publicly available, time-stamped textual feedback that may complement formal patient-experience surveys, complaint systems, and institutional quality-monitoring tools. For reviews expressing severe dissatisfaction, hospitals may consider prioritizing recurring and potentially avoidable communication failures along the care pathway, especially at stages where patients face high uncertainty and heavy navigation burdens. At the same time, the frequent co-occurrence of Efficiency/Process and Convenience/Environment suggests that process optimization and environmental support are not perceived as separate by patients. Queueing, registration, in-hospital signage, spatial layout, and on-site guidance often jointly shape patients' overall judgment of whether the service process is smooth. In addition, the large share of mixed sentiment among high-rating reviews suggests that online reviews may be particularly useful for capturing in-between experience signals that fall between traditional satisfaction surveys and formal complaints. Such issues are often not yet serious enough to enter formal complaint procedures, but may already be reflected in patient word of mouth, platform narratives, and subsequent patient expectations. In this sense, online patient reviews can serve as a supplementary data source beyond routine hospital monitoring for identifying recurrent service frictions. Even so, they should complement existing quality assessment systems and governance mechanisms rather than replace them. For hospital managers, such review-based domain mapping may help prioritize communication training, waiting-time management, registration flow, on-site guidance, and accessibility-related improvements, provided that review signals are interpreted alongside formal institutional data.

### Annotation and modeling approach

The annotation and modeling approach used in this study also has to be understood in relation to alternative methods. Sentence-level annotation, multiple-aspect annotation, and aspect-based sentiment analysis can provide more detailed information by identifying several aspects within the same comment and linking each aspect to a sentiment polarity. For example, recent work on Norwegian patient feedback used aspect-based sentiment analysis to code patient-experience comments at a finer level (58). In the present study, we used a review-level main-domain and secondary-domain approach because our aim was to produce an interpretable service-experience map rather than a fine-grained NLP benchmark. Our modeling strategy was also closer to a pipeline approach: domain and sentiment labels were first generated and then used in statistical models. Joint modeling approaches may capture relationships between aspects and sentiment more directly, but they are usually less transparent for applied hospital-quality interpretation. Thus, our approach is easier to review and interpret, but it may miss some within-review details and may allow annotation errors to carry forward into later analyses.

### Strengths and limitations

This study has several strengths. First, it used real-world public review data over a relatively long time span and therefore directly reflected service-experience content in patients' spontaneous expressions. Second, the study did not treat ratings, sentiment, and experience domains as the same thing; instead, it separately identified what patients were talking about and how they expressed it, allowing a more fine-grained description of the information contained in online reviews. Third, the coding framework was developed through preliminary reading, pilot coding, clinician discussion, and manual validation, while the LLM-assisted coding procedure was further supported by category-specific validation metrics and confusion matrices. Fourth, the analytical design considered contextual differences across campuses and assessed the statistical robustness of the findings across multiple statistical frameworks while keeping the overall interpretation appropriately conservative.

This study also has several limitations. First, online reviews are self-initiated data and do not represent all patients; more intense positive or negative experiences may be more likely to be expressed publicly. They may also overrepresent digitally active users and patients willing to share care experiences on public platforms, and the findings should not be interpreted as population-level estimates of patient-experience problems among all patients. Second, the reviews reflect patients' subjectively perceived service experiences rather than verified clinical events and are therefore better suited to identifying service frictions and perceived problems than to directly evaluating technical quality or clinical outcomes. Third, bringing campus- or hospital-level variables into review-level analysis inherently involves cross-level measurement, so even with statistical adjustment, the resulting estimates should still be interpreted conservatively. Fourth, large language model assistance was used in the annotation process. Although we attempted to reduce error through a structured prompt template, restricted output formats, and random human review, some misclassification may remain for reviews that were short, semantically ambiguous, or heavily dependent on specific context. This issue is better understood as defining the boundary of measurement precision rather than overturning the main association patterns observed here. The broad five-domain coding scheme is another limitation. Each domain was designed for macro-level service-experience mapping, but the categories are internally heterogeneous. For example, Convenience/Environment may include parking, crowding, cleanliness, wayfinding, spatial layout, and other access- or environment-related issues. These subthemes are not interchangeable and would require different improvement actions. Similarly, Efficiency/Process may include registration, waiting, examination flow, payment, medication dispensing, and interdepartmental navigation. Therefore, the five-domain framework is useful for identifying broad patterns in online reviews, but it is not sufficiently granular to serve as a direct checklist for hospital quality improvement. Future studies could combine this framework-level mapping with finer-grained coding, sentence-level annotation, or aspect-based sentiment analysis to identify more actionable subdomains (58). This study was not an inductive thematic analysis. Because no independent open-coding audit was conducted, recurrent out-of-framework, emerging, or context-specific themes may have been missed or underrepresented. Limiting each review to at most two domains may also have simplified highly multidimensional experiences.

The time-period grouping was based on sample distribution rather than policy milestones, and temporal patterns should therefore be interpreted cautiously. Parking-related POI counts were only a limited proxy for nearby parking-related facilities and did not capture capacity, fees, occupancy, walking distance, or on-site management conditions. The added sensitivity analyses assessed statistical robustness rather than the robustness of the conceptual coding framework, and online review patterns were not externally validated against patient surveys, complaint records, or hospital quality indicators. Fifth, the study used a cross-sectional observational design, so the findings should not be read as evidence of causality. Finally, the study focused on tertiary hospitals in a single city. Its findings are most applicable to similar urban tertiary-hospital and platform-review contexts rather than to all regions and all medical institutions.

The international relevance of this study lies mainly in its setting and use of free-text review data. Previous research has shown that online reviews can contain patient-experience information, but evidence from ordinary Chinese prefecture-level cities remains limited. By focusing on tertiary hospitals in Wuxi, this study adds evidence from a non-megacity Chinese health-system context, where tertiary hospitals remain important care-seeking destinations and public platform reviews may reflect service frictions across communication, process, perceived quality, cost transparency, and environment. The findings may be useful for international patient-centered care research by showing how free-text online reviews can be used as a complementary source of information alongside formal PREMs, complaint systems, and institutional quality indicators.

## Conclusion

Online patient reviews of tertiary hospitals in Wuxi showed interpretable domain patterns within a fixed, data-informed service-experience framework and highlighted several recurring patterns associated with low ratings, particularly those involving Attitude / Communication and negative or mixed text sentiment. Because ratings and narrative text do not carry identical information, online reviews may provide patient-generated signals for identifying perceived service frictions and locating issues relevant to process optimization, communication improvement, and patient-centered service monitoring. These findings should be interpreted as review-level associations under contextual adjustment and are most applicable to similar urban tertiary-hospital and platform-review contexts. Further external validation against formal PREMs, patient-experience surveys, complaint records, and hospital quality indicators is needed before such review-based patterns can be interpreted as independent indicators of hospital service quality.

## Data Availability

The data analyzed in this study were obtained from the Dianping platform and are subject to platform terms of use. The authors do not have permission to redistribute raw review text or identifiable user-level information. To support transparency and reproducibility, de-identified coding results, derived analytic datasets, statistical code, and LLM-assisted coding outputs may be made available by the corresponding author on reasonable request, subject to applicable platform terms and ethical considerations.
