# Supplementary material for: Mapping online patient reviews onto a data-informed service-experience framework: a content analysis of tertiary hospitals in Wuxi, China
Source: Front Public Health. 2026 Jul 13;14:1838026. doi: 10.3389/fpubh.2026.1838026 (PMC13408656; doi:10.3389/fpubh.2026.1838026)
Supplement: Supplementary file 1 [file Supplementary_file_1.docx]

# Supplementary material

Supplementary material for this article includes Supplementary Appendix 1, Supplementary Appendix 2, Supplementary Tables S1–S6, and Supplementary Figures S1–S2.

# Supplementary Appendix 1. Coding manual for patient-experience domains and text sentiment

## 1. Purpose and general principles

This study used a structured coding framework to classify Chinese online hospital reviews into patient-experience domains and overall text sentiment. The purpose of coding was to transform unstructured review text into analyzable variables reflecting the core experience content expressed by patients, rather than to verify medical facts or to assess the objective correctness of the review.

All judgments were based on the review text itself, not on the numerical platform rating. Coding followed a fixed sequence: 1. Determine the main patient-experience domain 2. Determine whether a distinct secondary domain was present 3. Determine the overall text sentiment

The coding framework was intended as an operational tool for structured analysis of patient-experience content in online reviews. It was not designed as a replacement for existing patient-experience theories or service-quality models.

## 2. Coding sequence

For each review, coding proceeded in the following order:

## Step 1. Main domain

Identify the single patient-experience domain that carried the primary evaluative meaning of the review.

## Step 2. Secondary domain

Determine whether the review also contained a second, distinct, and substantively meaningful experience domain.

## Step 3. Text sentiment

Based on the review text itself, classify the overall text sentiment as positive, negative, or mixed.

If the text did not contain enough evaluative information for a given task, the relevant field was marked as unable to determine.

## 3. Rules for main-domain assignment

The main domain was defined as the experience dimension that carried the main evaluative weight of the entire review.

If a review mentioned multiple dimensions, the main domain was determined by semantic importance, not by sentence order. The main domain should represent the dimension that best captured the patient's core praise or core complaint.

The following principles were used: - Select only one main domain for each review. - Do not determine the main domain based simply on which topic appeared first. - If multiple topics appeared, prioritize the one that contributed most to the overall evaluative meaning. - If the boundary between possible main and secondary domains was unclear, assign the domain corresponding to the review's primary praise or primary complaint as the main domain.

If no clear main domain could be determined from the text, the main-domain field was marked as unable to determine, and the review was excluded from domain-based analysis.

## 4. Rules for secondary-domain assignment

A secondary domain was assigned only when all of the following conditions were met: 1. The review clearly referred to a second domain that was different from the main domain 2. That second domain contained substantive evaluative content 3. The second domain was not merely a minor elaboration or supporting detail of the main domain

Only one secondary domain could be assigned per review.

A secondary domain should not be assigned when: - the second topic merely clarified or expanded the main domain - the second topic was mentioned only in passing - the second topic lacked clear evaluative meaning - the second topic was too weak to stand as an independent aspect of the review

If no eligible secondary domain was present, the secondary-domain field was recorded as none.

## 5. Definition of the five patient-experience domains

## 5.1 Attitude / Communication

### Definition

This domain covered comments about the attitudes, responsiveness, explanations, respectfulness, patience, listening behavior, or communication quality of physicians, nurses, registration staff, window staff, or other hospital personnel.

### Typical content

- willingness to explain
- politeness or rudeness
- patience or impatience
- whether questions were answered
- whether the patient felt respected or ignored
- adequacy of communication

### Positive examples

- "The doctor explained everything clearly and was willing to answer questions."
- "The nurse was patient and communicated well."

### Negative examples

- "The window staff were rude and impatient."
- "No one explained anything when I asked."

### Boundary reminders

- "I had to wait a long time before seeing the doctor" is generally Efficiency / Process
- "Parking was inconvenient" is generally Convenience / Environment

## 5.2 Efficiency / Process

### Definition

This domain covered comments on registration, queuing, waiting, appointment flow, examination scheduling, payment, medication pickup, referrals, calling systems, procedural coordination, and the overall efficiency of the care process.

### Typical content

- long waiting times
- repeated queuing
- complicated procedures
- repeated back-and-forth movement
- inefficient test or payment processes
- poor coordination between steps

### Positive examples

- "The examination process was smooth and I did not have to run around repeatedly."
- "The overall workflow was efficient."

### Negative examples

- "The queue was too long. Registration, payment, and examination all took too much time."
- "The process was complicated and poorly coordinated."

### Boundary reminders

- "The doctor was highly skilled" is generally Perceived Quality
- "Parking was difficult" is generally Convenience / Environment

## 5.3 Cost / Transparency

### Definition

This domain covered comments on the level of charges, reasonableness of costs, clarity of billing items, whether expenses were explained in advance, and perceived transparency of pricing or charges.

### Typical content

- charges too high
- unclear fee structure
- insufficient explanation of costs
- concern about value for money
- perceived lack of transparency

### Positive examples

- "There were many tests, but the charges were explained clearly."
- "The fees were reasonable and transparent."

### Negative examples

- "The costs were high and not explained beforehand."
- "The charges felt unclear."

### Boundary reminders

- "The process was complicated" is generally Efficiency / Process
- "The treatment did not work well" is generally Perceived Quality

## 5.4 Perceived Quality

### Definition

This domain covered the patient's subjective perception of physician competence, professionalism, diagnostic accuracy, treatment effectiveness, therapeutic outcome, and overall quality of care.

### Typical content

- doctor's skill or professionalism
- diagnostic accuracy
- whether treatment worked
- confidence in medical judgment
- perceived quality of care

### Positive examples

- "The physician was very professional and the treatment worked well."
- "The diagnosis seemed accurate and the care was effective."

### Negative examples

- "I saw the doctor several times but the problem was not solved."
- "It did not feel professional."

### Boundary reminders

- "The doctor was impatient" is generally Attitude / Communication
- "The procedures were too cumbersome" is generally Efficiency / Process

## 5.5 Convenience / Environment

### Definition

This domain covered comments on parking, transportation access, directions, signage, physical layout, hospital cleanliness, crowding, noise, basic facilities, and overall environmental comfort or convenience.

### Typical content

- parking availability
- campus navigation and signage
- cleanliness
- crowding
- environmental comfort
- ease of finding departments
- facility convenience

### Positive examples

- "The environment was clean and parking was convenient."
- "The signs were clear and it was easy to find the department."

### Negative examples

- "The campus signs were unclear and it was hard to find the clinic."
- "Parking was very inconvenient."

### Boundary reminders

- "The examination queue was slow" is generally Efficiency / Process
- "The charges were too high" is generally Cost / Transparency

## 6. Rules for text-sentiment classification

Text sentiment was judged from the review narrative itself, not from the platform rating.

## 6.1 Positive sentiment

A review was classified as positive when the overall message was mainly one of satisfaction, recognition, or praise, even if minor reservations were present, provided those reservations did not constitute the core negative content of the review.

### Example

- "The doctor was very careful and the overall experience was good."

## 6.2 Negative sentiment

A review was classified as negative when the overall message was mainly one of dissatisfaction, criticism, or complaint, even if polite softening language appeared.

### Example

- "The waiting time was too long and communication was poor. I would not come again."

## 6.3 Mixed sentiment

A review was classified as mixed when it contained both a clear positive element and a clear negative element, and both were central to the review's meaning.

Mixed sentiment was not: - a mostly positive review with a minor reservation - a mostly negative review with a polite or formulaic softening phrase

### Example

- "The doctor was competent, but the whole process was exhausting and the waiting time was too long."

If sentiment direction could not be determined from the text, the sentiment field was marked as unable to determine, and the review was excluded from sentiment analysis.

## 7. Exclusion from specific analyses

A review was not included in the corresponding analysis when it met one of the following conditions:

## 7.1 Exclusion from domain analysis

- only symbols, emojis, or non-semantic text
- extremely short text with no clear experience content
- purely factual statements without evaluative meaning
- no clear main domain could be identified
- the text was insufficient to support structured domain classification

## 7.2 Exclusion from sentiment analysis

- no clear evaluative direction
- purely factual statements
- semantic information insufficient to judge sentiment
- sentiment could not be determined under the coding rules

A review could be excluded from one analysis but still be eligible for another if the relevant field could be determined.

## 8. Anonymous example reviews

## Example 1

### Review

"The doctor explained everything in detail and the treatment was effective, but the queue was too long."

### Main domain

Perceived Quality

### Secondary domain

Efficiency / Process

### Text sentiment

Mixed

## Example 2

### Review

"The registration window staff spoke very harshly and no one explained anything."

### Main domain

Attitude / Communication

### Secondary domain

None

### Text sentiment

Negative

## Example 3

### Review

"The hospital environment was nice and the route signs were clear."

### Main domain

Convenience / Environment

### Secondary domain

None

### Text sentiment

Positive

## Example 4

### Review

"I had to go back and forth for examinations many times. The process was not smooth."

### Main domain

Efficiency / Process

### Secondary domain

None

### Text sentiment

Negative

## Example 5

### Review

"The charges were a bit high, but the doctor was still quite responsible."

### Main domain

Cost / Transparency

### Secondary domain

Attitude / Communication

### Text sentiment

Mixed

## Example 6

### Review

"The specialist was very professional, but the directions were unclear and parking was inconvenient."

### Main domain

Perceived Quality

### Secondary domain

Convenience / Environment

### Text sentiment

Mixed

## Example 7

### Review

"It was okay."

### Main domain

Unable to determine

### Secondary domain

None

### Text sentiment

Unable to determine

### Handling

Excluded from both domain and sentiment analyses.

## Example 8

### Review

"Returned for a follow-up visit today and got medication."

### Main domain

Unable to determine

### Secondary domain

None

### Text sentiment

Unable to determine

### Handling

Factual record only; excluded from sentiment analysis and domain analysis.

# Supplementary Appendix 2. Structured prompt template for LLM-assisted coding

## 1. Purpose

The large language model was used only to assign structured labels according to the prespecified coding manual. It was not used to generate new themes, revise the coding framework, or provide open-ended summaries or interpretations.

## 2. Input unit

Each input consisted of one original Chinese patient review.

## 3. Task objective

For each review, complete the following tasks in order: 1. Determine the single main patient-experience domain 2. Determine whether a distinct and substantively meaningful secondary domain is present 3. Determine the overall text sentiment 4. If the text does not provide enough information for a clear judgment, mark the relevant field as unable to determine 5. Indicate whether the review should be included in domain analysis and sentiment analysis

## 4. Required processing order

All reviews must be processed in the following order: 1. Main domain 2. Secondary domain 3. Text sentiment

Do not change this sequence.

## 5. Allowed labels

## 5.1 Main domain / secondary domain

- Attitude / Communication
- Efficiency / Process
- Cost / Transparency
- Perceived Quality
- Convenience / Environment

## 5.2 Text sentiment

- Positive
- Negative
- Mixed
- Unable to determine

## 5.3 Secondary-domain special value

- None

## 6. Decision rules

## 6.1 Main-domain assignment

Identify the domain that carries the primary evaluative meaning of the review. If multiple domains are mentioned, choose the one that best captures the review's core praise or core complaint. Do not determine the main domain simply by sentence order.

If the boundary between possible main and secondary domains is unclear, assign as the main domain the dimension corresponding to the review's primary praise or primary complaint.

If no clear main domain can be identified, mark the main domain as unable to determine and set Include in domain analysis = No.

## 6.2 Secondary-domain assignment

Assign a secondary domain only when: - it is clearly different from the main domain - it has substantively meaningful evaluative content - it is not merely a minor elaboration of the main domain

Assign at most one secondary domain.

If no eligible secondary domain is present, record Secondary domain = None.

## 6.3 Sentiment assignment

Classify sentiment based on the review text only, not on the numerical platform rating. - Positive: overall satisfaction, praise, or recognition; minor reservations do not change the main positive direction - Negative: overall dissatisfaction, criticism, or complaint; polite softening language does not change the main negative direction - Mixed: both a clear positive element and a clear negative element are present, and both are central to the review

Mixed is not: - a mainly positive review with a slight reservation - a mainly negative review with a formulaic polite phrase

If sentiment direction cannot be judged clearly, record Text sentiment = Unable to determine and set Include in sentiment analysis = No.

## 6.4 Exclusion logic

- If no clear main domain can be identified, do not include the review in domain analysis
- If no clear sentiment can be identified, do not include the review in sentiment analysis
- A review may be included in one analysis and excluded from the other, depending on which field can be determined

## 7. Decision constraints

Follow all of the following constraints: - Use the review text only - Do not infer from the numerical rating - Assign only one main domain - Assign at most one secondary domain - Do not create new labels - Do not output explanations, summaries, or free text - If the text is too short, factual only, semantically insufficient, or lacks evaluative meaning, mark the relevant field as unable to determine

## 8. Required output schema

Return the result in the following format only: - Main domain: - Secondary domain: - Text sentiment: - Include in domain analysis: Yes / No - Include in sentiment analysis: Yes / No

Do not output anything else.

## 9. Reproducible prompt template

You are assisting with structured coding of Chinese hospital review texts.

Follow the coding manual strictly. Do not create new labels. Use only the allowed labels listed below. Your task is not to generate new themes, but to assign structured labels within the prespecified framework.

Process each review in this order: 1. Determine the main domain 2. Determine whether a distinct secondary domain is present 3. Determine the overall text sentiment

Use the review text itself rather than the numerical rating.

Main-domain rule: Identify the domain that carries the primary evaluative meaning of the review. If multiple domains are mentioned, choose the one that best captures the review's core praise or core complaint. If the main/secondary boundary is unclear, use the domain corresponding to the primary praise or primary complaint as the main domain. If no clear main domain can be determined, mark it as "Unable to determine".

Secondary-domain rule: Assign a secondary domain only if it is clearly distinct from the main domain and contains substantively meaningful evaluative content. If it only slightly elaborates the main domain, do not assign it. Assign at most one secondary domain. If none is present, return "None".

Sentiment rule: Classify the review as Positive, Negative, or Mixed based on the review text itself. Mixed means that both a clear positive element and a clear negative element are present and both are central to the review. Mixed does not mean a mainly positive review with a minor reservation, or a mainly negative review with a polite phrase. If sentiment cannot be determined, mark it as "Unable to determine".

Allowed main/secondary domains: - Attitude / Communication - Efficiency / Process - Cost / Transparency - Perceived Quality - Convenience / Environment

Allowed sentiment labels: - Positive - Negative - Mixed - Unable to determine

Output only in the following format: - Main domain: - Secondary domain: - Text sentiment: - Include in domain analysis: Yes / No - Include in sentiment analysis: Yes / No

Review text: "[Insert original Chinese review here]"

## 10. Example output

- Main domain: Perceived Quality
- Secondary domain: Efficiency / Process
- Text sentiment: Mixed
- Include in domain analysis: Yes
- Include in sentiment analysis: Yes

# Supplementary Table S1. Comparison of the fully adjusted model across the primary clustered logistic regression, mixed-effects logistic regression, and GEE logistic regression frameworks

| **Variable** | **Main analysis OR (95% CI)** | **Main analysis P value** | **Mixed-effects OR (95% CI)** | **Mixed-effects P value** | **GEE OR (95% CI)** | **GEE P value** |
| --- | --- | --- | --- | --- | --- | --- |
| Attitude / Communication | 2.92 (1.86, 4.58) | <0.001 | 2.92 (2.08, 4.10) | <0.001 | 2.92 (1.90, 4.48) | <0.001 |
| Efficiency / Process | 0.60 (0.43, 0.84) | 0.002 | 0.60 (0.44, 0.82) | 0.001 | 0.60 (0.44, 0.82) | 0.002 |
| Cost / Transparency | 1.60 (0.95, 2.68) | 0.079 | 1.60 (0.92, 2.76) | 0.096 | 1.59 (0.97, 2.62) | 0.066 |
| Convenience / Environment | 0.36 (0.26, 0.51) | <0.001 | 0.36 (0.23, 0.58) | <0.001 | 0.36 (0.26, 0.50) | <0.001 |
| Mixed sentiment | 4.28 (3.08, 5.93) | <0.001 | 4.28 (3.01, 6.08) | <0.001 | 4.28 (3.13, 5.86) | <0.001 |
| Negative sentiment | 14.64 (10.37, 20.68) | <0.001 | 14.64 (9.96, 21.54) | <0.001 | 14.71 (10.60, 20.40) | <0.001 |
| Secondary domain present | 0.79 (0.66, 0.95) | 0.012 | 0.79 (0.61, 1.01) | 0.065 | 0.79 (0.66, 0.95) | 0.010 |
| 2014–2017 period | 0.91 (0.66, 1.27) | 0.594 | 0.91 (0.70, 1.20) | 0.515 | 0.90 (0.66, 1.24) | 0.525 |
| 2009–2013 period | 1.46 (0.58, 3.66) | 0.424 | 1.46 (0.95, 2.22) | 0.082 | 1.44 (0.59, 3.50) | 0.425 |
| Specialty tertiary | 0.72 (0.51, 1.01) | 0.059 | 0.72 (0.54, 0.97) | 0.032 | 0.72 (0.55, 0.93) | 0.014 |
| Low parking availability | 1.18 (0.85, 1.62) | 0.320 | 1.18 (0.88, 1.58) | 0.272 | 1.18 (0.93, 1.50) | 0.176 |

Note: All estimates shown here correspond to the fully adjusted specification (Model 2).

Main analysis: ordinary logistic regression with campus-clustered robust standard errors.

Mixed-effects analysis: logistic regression with a campus random intercept.

GEE analysis: logistic regression with campus as the clustering unit and an exchangeable working correlation structure.

# Supplementary Table S2. Validation metrics and confusion matrices for LLM-assisted coding

**Panel A. Category-specific validation metrics for primary experience-domain coding**

| **Category** | **Precision** | **Recall** | **F1-score** | **Support** |
| --- | --- | --- | --- | --- |
| Attitude / Communication | 0.735 | 0.833 | 0.781 | 30 |
| Efficiency / Process | 0.913 | 0.875 | 0.894 | 72 |
| Cost / Transparency | 0.667 | 0.750 | 0.706 | 8 |
| Perceived Quality | 0.896 | 0.878 | 0.887 | 49 |
| Convenience / Environment | 0.925 | 0.902 | 0.914 | 41 |
| Overall accuracy |  |  | 0.870 | 200 |
| Macro average | 0.827 | 0.848 | 0.836 |  |
| Weighted average | 0.875 | 0.870 | 0.872 | 200 |

**Panel B. Confusion matrix for primary experience-domain coding**

| Human consensus reference label | Attitude / Communication | Efficiency / Process | Cost / Transparency | Perceived Quality | Convenience / Environment |
| --- | --- | --- | --- | --- | --- |
| Attitude / Communication | 25 | 2 | 2 | 1 | 0 |
| Efficiency / Process | 4 | 63 | 1 | 2 | 2 |
| Cost / Transparency | 2 | 0 | 6 | 0 | 0 |
| Perceived Quality | 2 | 3 | 0 | 43 | 1 |
| Convenience / Environment | 1 | 1 | 0 | 2 | 37 |

**Panel C. Validation metrics for secondary-domain presence/absence**

| **Task** | **Accuracy** | **Present precision** | **Present recall** | **Present F1-score** | **Support** |
| --- | --- | --- | --- | --- | --- |
| Secondary-domain presence/absence | 0.880 | 0.852 | 0.920 | 0.885 | 200 |

**Panel D. Confusion matrix for secondary-domain presence/absence**

| Human consensus reference label \ Initial LLM-assisted label | Absent | Present |
| --- | --- | --- |
| Absent | 84 | 16 |
| Present | 8 | 92 |

**Panel E. Category-specific validation metrics for secondary experience-domain coding**

| **Category** | **Precision** | **Recall** | **F1-score** | **Support** |
| --- | --- | --- | --- | --- |
| Attitude / Communication | 0.923 | 0.900 | 0.911 | 40 |
| Efficiency / Process | 0.936 | 0.898 | 0.917 | 49 |
| Cost / Transparency | 0.789 | 0.882 | 0.833 | 17 |
| Perceived Quality | 0.886 | 0.907 | 0.897 | 43 |
| Convenience / Environment | 0.902 | 0.902 | 0.902 | 51 |
| Overall accuracy |  |  | 0.900 | 200 |
| Macro average | 0.887 | 0.898 | 0.892 |  |
| Weighted average | 0.902 | 0.900 | 0.900 | 200 |

**Panel F. Confusion matrix for secondary experience-domain coding**

| Human consensus reference label | Attitude / Communication | Efficiency / Process | Cost / Transparency | Perceived Quality | Convenience / Environment |
| --- | --- | --- | --- | --- | --- |
| Attitude / Communication | 36 | 1 | 0 | 2 | 1 |
| Efficiency / Process | 1 | 44 | 2 | 1 | 1 |
| Cost / Transparency | 0 | 0 | 15 | 1 | 1 |
| Perceived Quality | 2 | 0 | 0 | 39 | 2 |
| Convenience / Environment | 0 | 2 | 2 | 1 | 46 |

**Panel G. Category-specific validation metrics for text-sentiment coding**

| **Category** | **Precision** | **Recall** | **F1-score** | **Support** |
| --- | --- | --- | --- | --- |
| Positive | 0.922 | 0.865 | 0.892 | 96 |
| Negative | 0.873 | 0.838 | 0.855 | 74 |
| Mixed | 0.641 | 0.833 | 0.725 | 30 |
| Overall accuracy |  |  | 0.850 | 200 |
| Macro average | 0.812 | 0.845 | 0.824 |  |
| Weighted average | 0.862 | 0.850 | 0.853 | 200 |

**Panel H. Confusion matrix for text-sentiment coding**

| Human consensus reference label | Positive | Negative | Mixed |
| --- | --- | --- | --- |
| Positive | 83 | 6 | 7 |
| Negative | 5 | 62 | 7 |
| Mixed | 2 | 3 | 25 |

Note: Post-adjudication human consensus labels were used as the reference standard, and initial LLM-assisted labels were treated as predicted labels. Precision, recall, F1-score, and support were calculated for each category. Macro average represents the unweighted mean of category-specific metrics, whereas weighted average represents the support-weighted mean. Confusion matrices are presented with rows indicating human consensus reference labels and columns indicating initial LLM-assisted labels. Secondary-domain validation was evaluated in two steps. First, the binary presence/absence of a clear secondary domain was assessed because this variable was used in the regression model. Second, category classification was evaluated in the secondary-domain category-validation subset, which consisted of reviews with a clear secondary domain. Metrics for low-frequency categories, particularly Cost / Transparency, should be interpreted cautiously because precision, recall, and F1-scores may be unstable when validation counts are small.

# Supplementary Table S3. Ordinal regression sensitivity analysis

| **Variable** | **Ordinal OR (95% CI)** | **P value** |
| --- | --- | --- |
| Attitude / Communication | 0.40 (0.30, 0.53) | <0.001 |
| Efficiency / Process | 1.40 (1.09, 1.79) | 0.008 |
| Cost / Transparency | 0.70 (0.44, 1.13) | 0.145 |
| Convenience / Environment | 1.59 (1.18, 2.15) | 0.002 |
| Mixed sentiment | 0.32 (0.26, 0.41) | <0.001 |
| Negative sentiment | 0.10 (0.07, 0.13) | <0.001 |
| Secondary domain present | 1.27 (1.04, 1.54) | 0.017 |
| 2014-2017 period | 0.45 (0.37, 0.55) | <0.001 |
| 2009-2013 period | 0.29 (0.21, 0.41) | <0.001 |
| Specialty tertiary | 1.38 (1.09, 1.74) | 0.007 |
| Low parking availability | 0.97 (0.77, 1.23) | 0.822 |

Note: Ordinal odds ratios greater than 1 indicate higher odds of being in a higher ordered rating category (low < middle < high). Therefore, estimates in the opposite direction to the binary low-rating model indicate directional consistency. The model used the same covariate structure as the fully adjusted primary model. A cumulative-logit diagnostic was used to examine directional consistency across rating thresholds.

# Supplementary Table S4. Logistic regression excluding sentiment

| **Variable** | **Excluding sentiment OR (95% CI)** | **P value** |
| --- | --- | --- |
| Attitude / Communication | 2.87 (1.83, 4.52) | <0.001 |
| Efficiency / Process | 0.84 (0.59, 1.20) | 0.345 |
| Cost / Transparency | 2.16 (1.46, 3.20) | <0.001 |
| Convenience / Environment | 0.32 (0.23, 0.46) | <0.001 |
| Secondary domain present | 0.67 (0.57, 0.80) | <0.001 |
| 2014-2017 period | 0.97 (0.72, 1.30) | 0.825 |
| 2009-2013 period | 1.61 (0.70, 3.68) | 0.260 |
| Specialty tertiary | 0.74 (0.56, 0.99) | 0.042 |
| Low parking availability | 1.13 (0.82, 1.54) | 0.458 |

Note: Odds ratios greater than 1 indicate higher odds of low-rating reviews. This sensitivity model retained the primary binary logistic model covariates except text sentiment, which was excluded because text sentiment and numerical rating are conceptually overlapping evaluative constructs.

# Supplementary Table S5. Descriptive comparison between the full analytic sample and the 200-review LLM-output validation subset

| **Characteristic** | **Full analytic sample, n (%)** | **LLM-output validation subset, n (%)** |
| --- | --- | --- |
| **Rating group** |  |  |
| **Low rating** | **407 (17.1%)** | **43 (21.5%)** |
| Middle rating | 337 (14.2%) | 26 (13.0%) |
| High rating | 1637 (68.8%) | 131 (65.5%) |
| **Time period** |  |  |
| 2009–2013 | 160 (6.7%) | 11 (5.5%) |
| 2014–2017 | 738 (31.0%) | 72 (36.0%) |
| 2018–2025 | 1483 (62.3%) | 117 (58.5%) |
| **Hospital type** |  |  |
| General tertiary | 1313 (55.1%) | 113 (56.5%) |
| Specialty tertiary | 1068 (44.9%) | 87 (43.5%) |
| **Main experience domain** |  |  |
| Attitude / Communication | 364 (15.3%) | 44 (22.0%) |
| Efficiency / Process | 858 (36.0%) | 66 (33.0%) |
| Cost / Transparency | 92 (3.9%) | 12 (6.0%) |
| Perceived Quality | 580 (24.4%) | 43 (21.5%) |
| Convenience / Environment | 487 (20.5%) | 35 (17.5%) |

Note: The full analytic sample included 2,381 eligible reviews, whereas the LLM-output validation subset included 200 reviews sampled for post-adjudication validation of initial LLM-assisted labels. Percentages were calculated within each column. The validation subset was descriptively compared with the full analytic sample to assess coverage across key review characteristics. Because some categories, particularly low-frequency categories, contained relatively small validation counts, category-specific validation metrics should be interpreted cautiously.

# Supplementary Table S6. Distribution of original Dianping rating categories

| **Original platform rating** | **n (%)** |
| --- | --- |
| 0.5 | 104 (4.37%) |
| 1.0 | 177 (7.43%) |
| 1.5 | 17 (0.71%) |
| 2.0 | 96 (4.03%) |
| 2.5 | 13 (0.55%) |
| 3.0 | 337 (14.15%) |
| 3.5 | 48 (2.02%) |
| 4.0 | 765 (32.13%) |
| 4.5 | 150 (6.30%) |
| 5.0 | 674 (28.31%) |
| **Total** | **2,381 (100.00%)** |

Note: Percentages were calculated using all eligible reviews included in the analytic sample as the denominator (n = 2,381). Low-, middle-, and high-rating groups were defined as 0.5–2.5, 3.0, and 3.5–5.0, respectively.

# Supplementary Figure S1. Forest plot of the fully adjusted mixed-effects logistic regression model


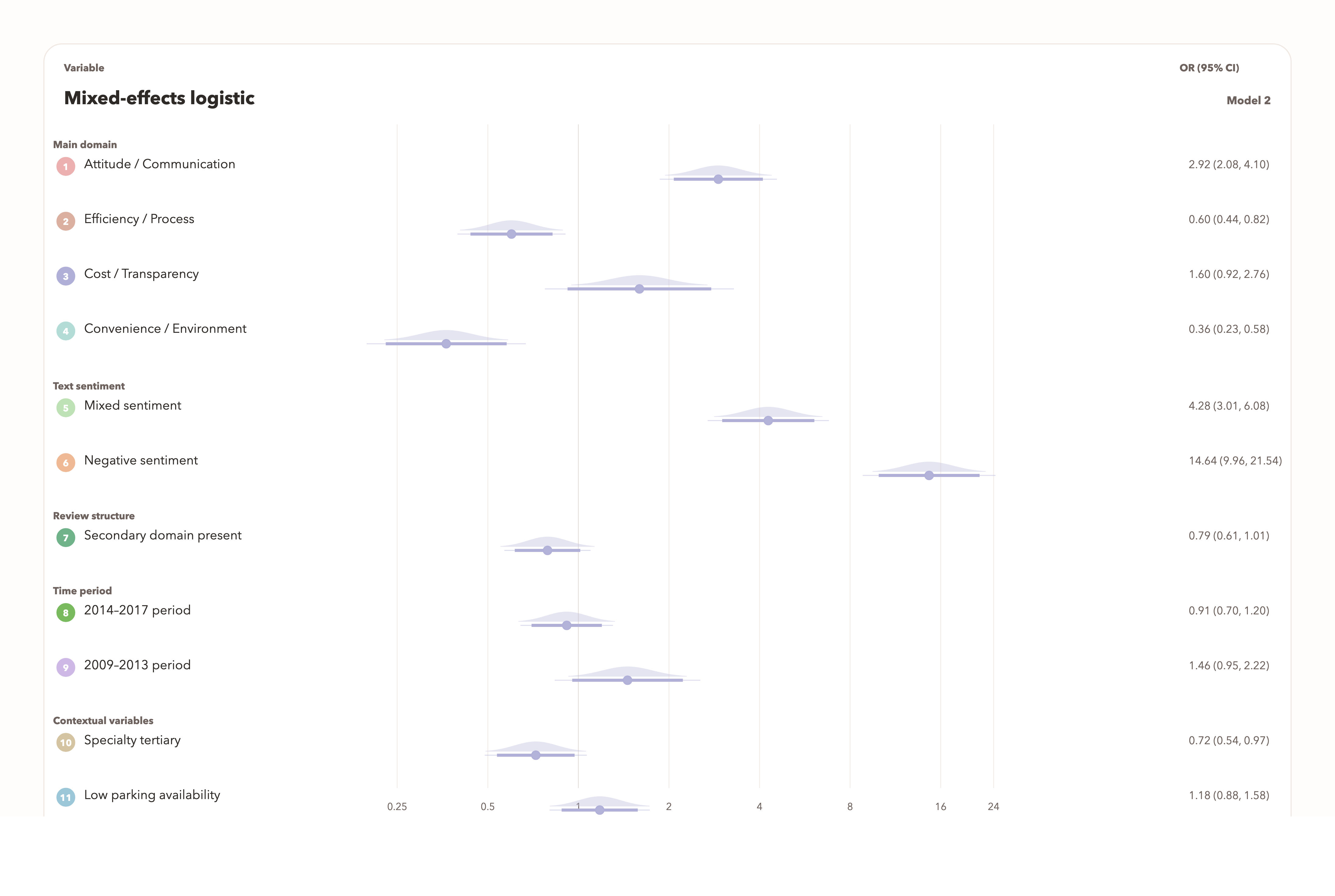


Caption: Estimates were obtained from a review-level mixed-effects logistic regression model with a random intercept for campus. Points represent odds ratios (ORs), and horizontal lines represent 95% confidence intervals (CIs). All results are interpreted as associations rather than causal effects.

# Supplementary Figure S2. Forest plot of the fully adjusted GEE logistic regression model


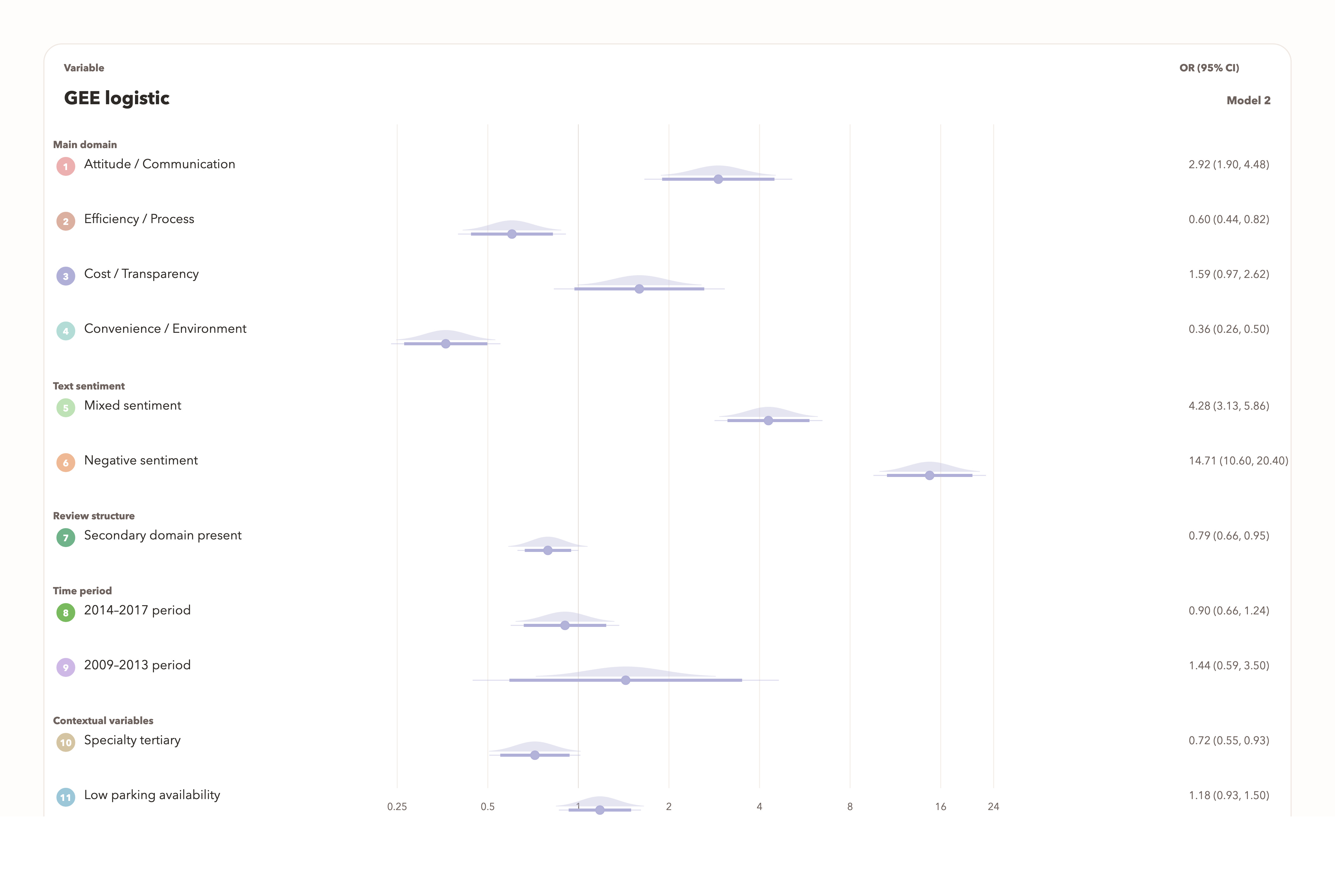


Caption: Estimates were obtained from a review-level GEE logistic regression model clustered by campus with an exchangeable working correlation structure. Points represent odds ratios (ORs), and horizontal lines represent 95% confidence intervals (CIs). All results are interpreted as associations rather than causal effects.
